# Supplementary material for: Study to evaluate the effectiveness and cost-effectiveness of different screening strategies for identifying undiagnosed COPD among residents (≥40 years) in four cities in China: protocol for a multicentre cross-sectional study on behalf of the Breathe Well group
Source: BMJ Open. 2020 Nov 27;10(11):e035738. doi: 10.1136/bmjopen-2019-035738 (PMC7703419; doi:10.1136/bmjopen-2019-035738)
Supplement: Supplementary data [file bmjopen-2019-035738supp001.pdf]

Supplemental material BMJ Publishing Group Limited (BMJ) disclaims all liability and responsibility arising from any reliance placed on this supplemental material which has been supplied by the author(s) BMJ Open

Evaluating screening strategies for identifying undiagnosed COPD in China: a Breathe Well project

中国慢阻肺筛查策略评估：健康呼吸 Breathe Well 研究项目

Lung health questionnaire

肺部健康问卷

Participant Initials<br>研究对象编号	
Study ID<br>问卷编号	
Date<br>填写日期	
Interviewer ID<br>研究人员编号	

Pan Z, *et al. BMJ Open* 2020; 10:e035738. doi: 10.1136/bmjopen-2019-035738

Some questions in the following booklets may appear similar. However, it is important that we ask these questions in slightly different ways so please complete all questions, answering them as accurately as possible.

一些问题可能相似，但是我们以稍微不同的方式提出这些问题很重要。

因此，请您完成所有的问题，并尽可能准确地作答。

CDQ

1. Age group, years
   年龄

40–49 ☐ 50-59 ☐ 60-69 ☐ 70+ ☐

2. What is your weight in kilograms?
   您的体重（公斤）？

   ______________kilograms
   ______________ 公斤

   What is your height in meters?
   您的身高（米）？

   ______________ metres
   ______________ 米

3. Smoking
   吸烟强度，包年
   What is the total number of years you have smoked?
   您一共吸烟多少年？
   ______ years

   _____ 年

   How many cigarettes do you currently smoke each day (or 'did smoke each day' if ex-smoker)?
   目前您每天吸多少支烟？（或，如果是既往吸烟者，过去您每天吸多少支烟？）
   ______ cigarettes

   _____ 支

4. Does the weather affect your cough?
   您的咳嗽是否受天气影响？

Yes ☐ No ☐

是 ☐ 否 ☐

5. Do you ever cough up phlegm (sputum) from your chest when you don’t have a cold?
   您不感冒的时候，会从胸腔里咳出痰吗？（区别于从嗓子中咳痰）

Yes ☐ No ☐
是 ☐ 否 ☐

6. Do you usually cough up phlegm (sputum) from your chest first thing in the morning?
   清晨您的第一件事是从胸腔里咳出痰吗？

Yes ☐ No ☐
是 ☐ 否 ☐

7. How frequently do you wheeze?
   您喘息的次数是多少？

Occasionally or more often ☐ Never ☐
有时候或更频繁 ☐ 从不 ☐

8. Do you have or have you had any allergies?
   目前或既往您有过敏物吗？

Yes ☐ No ☐
是 ☐ 否 ☐

CAPTURE

1. Have you ever lived or worked in a place with dirty or polluted water or air, smoke or second-hand smoke or dust?
   您是否曾经在有脏的或受到污染的水或空气，烟雾或二手烟雾或灰尘的地方生活或工作？

Yes ☐ No ☐
是 ☐ 否 ☐

2. Does your breathing change with seasons, weather or air quality?
   您的呼吸是否随着季节、天气或空气质量而变化？

Yes ☐ No ☐
是 ☐ 否 ☐

3. Does your breathing make it difficult to do things such as carry heavy loads, shovel dirt or snow, jog, play tennis or swim?
   您的呼吸是否会使您难以进行一些工作，比如提重物，铲土或积雪，慢跑，打网球或游泳等？

Yes ☐ No ☐
是 ☐ 否 ☐

4. Compared to others your age, do you tire easily?
和您的同龄人相比，您是否容易感到疲劳？

Yes ☐ No ☐
是 ☐ 否 ☐

5. In the past 12 months, how many times did you miss work, school, or other activities due to a cold, bronchitis, or pneumonia?
在过去的 12 个月里，您有多少次因感冒、支气管炎或肺炎而错过了工作、学校或其他活动？

0 ☐ 1 ☐ 2 or more ☐
0 ☐ 1 ☐ 2 或以上 ☐

Copyright© 2015 by Cornell University, University of Kentucky, and Evidera. All Rights Reserved
版权所有©2015 康奈尔大学，肯塔基大学和 Evidera。版权所有

Symptom-based questionnaire

1. How frequently are you exposed to second-hand smoking?
您接触二手烟的频率是多少？

<7hrs per week ☐ ≥7hrs per week ☐

＜7小时/周 ☐ ＞7 小时/周 ☐

2. Do you often cough when you do not have a cold?
您是否在不感冒的时候经常咳嗽？

Yes ☐ No ☐
是 ☐ 否 ☐

3. Do you have more signs of shortness of breath compared with others of the same age?
和同龄人相比，您是否有更多的呼吸急促的症状？

Yes ☐ No ☐
是 ☐ 否 ☐

4. Have you had long-term exposure to dust or chemical particles?
您是否长期地接触粉尘或化学颗粒？

Yes ☐ No ☐

是 ☐ 否 ☐

5. Did you have a history of chronic respiratory diseases when you were a child?
在您孩童时期，您是否有慢性呼吸疾病的病史？

Yes ☐是 No ☐
是 ☐ 否 ☐

COPD-SQ

1. Do you often cough?
您是否经常咳嗽？

Yes ☐ No ☐
是 ☐ 否 ☐

2. Family history of respiratory disease
是否有呼吸疾病家族史？

Yes ☐ No ☐
是 ☐ 否 ☐

3. Exposure to biomass smoke from cooking fires
是否接触烹饪产生的生物烟雾？

Yes ☐ No ☐
是 ☐ 否 ☐
