# Supplementary material for: Study to evaluate the effectiveness and cost-effectiveness of different screening strategies for identifying undiagnosed COPD among residents (≥40 years) in four cities in China: protocol for a multicentre cross-sectional study on behalf of the Breathe Well group
Source: BMJ Open. 2020 Nov 27;10(11):e035738. doi: 10.1136/bmjopen-2019-035738 (PMC7703419; doi:10.1136/bmjopen-2019-035738)
Supplement: Supplementary data [file bmjopen-2019-035738supp002.pdf]

中国慢阻肺筛查策略评估：健康呼吸 Breathe Well 研究项目

Evaluating screening strategies for identifying undiagnosed COPD in China: a Breathe Well project

调查问卷

Study Questionnaire

研究对象编号<br>Patient Initials	
问卷编号<br>Study ID	
填写日期<br>Date	
研究人员编号<br>Interviewer ID	

您的回答和意见对们很有价值。请您在翻页之前阅读以下内容，非常感谢您的合作！

Your answers and opinions are valuable to us. We would be very grateful if you could read the below before turning the page:

- **如有可能，请您自行填写这份问卷。**

  Please complete this questionnaire yourself if at all possible
- **请尽可能回答所有问题**

  Please answer all questions as well as you can
- **请不要花太多时间思考您的回答**

  Do not spend too long thinking about your answers
- **如果有人替您回答了这份问卷，他们需要记录下您的答案**

  If someone is completing this on your behalf, they should record your answers

BREATHE WELL
BUILDING RESEARCH ACROSS THE WORLD IN LUNG DISEASE

调查问卷 v7 2018.10.4
Study questionnaire 041018

1. 性别

Sex

☐ 男

Male

☐ 女

Female

2. 年龄

What is your age?

岁

___________ years old

3. 您获得的最高学历是什么？

What is the highest level of qualification that you have?

没有正式的学历 ☐

No formal qualification

低于高中水平 ☐

Less than High school

高中水平 ☐

High school

大专

Junior college

本科 ☐

Bachelor

研究生 ☐

Master

博士 ☐

Doctor

4. 您的工作状态是什么样的？

What is your employment status?

调查问卷 v7 2018.10.4

Study questionnaire 041018

个体 ☐
Self-employed

受雇于工作单位 ☐
Employed

无工作 ☐
Unemployed

退休 ☐
Retired

5. 您绝大部分时间生活在哪里?
Where have you spent most of your life?

城市 ☐
Urban areas

农村 ☐
Rural areas

6. 您目前的吸烟状态是?
What is your current smoking status?

当前吸烟者(每天至少吸 1 支,至少吸了 6 个月) ☐
Current smoker (smoke at least 1 cigarette per day for at least the last 6 months)

既往吸烟者(既往每天至少吸 1 支,至少吸了 6 个月,但是现在不吸了) ☐
Ex-smoker (previously smoked at least 1 cigarette per day for at least 6 months, but not now)

我从不经常性地吸烟 (**如果您选择了这个选项,请跳至第 9 题**) ☐
I have never smoked regularly (***please go to question 10***)

7. 如果您曾经吸过烟,那么您是几岁**开始**经常性地吸烟?(“经常性地吸烟”指的是,至少 1 支/每天或者 7 支/每周,至少 6 个月)
If you have ever smoked, at about what age did you **start** to smoke regularly? (by regularly we mean at least 1 cigarette/day or 7 cigarettes/week for at least 6 months)
________ 岁

调查问卷 v7 2018.10.4
Study questionnaire 041018

_________ years old

如果您曾经吸过烟，您是从什么时候**停止**经常性地吸烟的？

If you are an ex-smoker, at what age did you **stop** smoking regularly?

_________ 岁

_________ years old

8．目前您每天常常吸多少支烟？或者，当您是烟民的时候，您是否经常性地吸烟？

How much do you usually smoke each day now, or did you usually smoke when you were a smoker?

电子烟<br>Electronic cigarettes (or e-cigarettes)		支/天<br>number/day
过滤嘴型香烟<br>Filter cigarettes		支/天<br>number/day
无过滤嘴/手卷烟<br>Non-filter/hand rolled cigarettes		支/天<br>number/day
雪茄<br>Cigars		支/天<br>number/day
烟斗<br>Pipe tobacco		烟草…克/天<br>g/day tobacco

9．您的整体健康状况如何?

How is your health in general?

非常好 Very Good☐ 好 Good☐ 一般 Fair☐ 差 Bad☐ 非常差 Very Bad☐

10．患病情况

Medical conditions

您患有以下疾病吗？请选择

Has a doctor EVER told you that you had any of the following conditions? Please tick all that apply

疾病<br>Conditions	有<br>Yes	无<br>No
慢性阻塞性肺疾病<br>Chronic Obstructive Pulmonary Disease		
慢性支气管炎/肺气肿<br>Chronic bronchitis/emphysema		

调查问卷 v7 2018.10.4
Study questionnaire 041018

哮喘<br>Asthma		
结核<br>Tuberculosis		
高血压<br>Hypertension		
糖尿病<br>Diabetes Mellitus		
胃食管返流<br>GERD		
焦虑<br>Anxiety		
抑郁<br>Depression		
心脏病<br>Heart disease		
癌症<br>Cancer		

11. 当您在水平地面上行走或在一个小山坡上行走时，您是否因呼吸急促而感到困扰？
Are you troubled by shortness of breath when hurrying on the level ground or walking up a slight hill?
是 Yes ☐ 否 No ☐

12. 您在平地上和同龄人一起行走时，您是否会感到气促？
Do you get short of breath walking with other people of your own age on level ground?
是 Yes ☐ 否 No ☐

13. 当您在平地上按自己的速度行走时，您是否会因为呼吸而不得不停下来？
Do you have to stop for breath when walking at your own pace on level ground?
是 Yes ☐ 否 No ☐

14. 当您在平地上行走 100 米或几分钟后，您是否会因为呼吸而不得不停下来？
Do you have to stop for breath after walking for 100yds (or after a few minutes) on the level?
是 Yes ☐ 否 No ☐

15. 您是否因呼吸困难而不能离开家或者您是否在穿衣服或脱衣服的时候有呼吸困难？
Are you too breathless to leave the house or are you breathless when dressing or undressing?
是 Yes ☐ 否 No ☐

调查问卷 v7 2018.10.4
Study questionnaire 041018

COPD 评估 (CAT)
COPD Assessment Test (CAT)

即使您没有肺部问题，也请完成以下问卷。
Please complete the below questionnaire even if you do not have a lung condition

16. 您肺部的问题怎么样？对于下面的每个项目，请在 0-5 中圈出最符合您的情况的分数。
How are your lung problems? For each item below place a mark in the box that best describes your experience on a scale of 0-5

例如：我极开心	0	✓ 1	2	3	4	5	我极不开心

Example: I am very happy | 0 | √ 1 | 2 | 3 | 4 | 5 | I am very sad

我从不咳嗽<br>I never cough	0	1	2	3	4	5	我总是咳嗽<br>I cough all the time
我肺里一点痰也没有<br>I have no phlegm (mucus) in my chest at all	0	1	2	3	4	5	我肺里有很多很多痰<br>My chest is completely full of phlegm (mucus)
我一点也没有胸闷的感觉<br>My chest does not feel tight at all	0	1	2	3	4	5	我有很重的胸闷的感觉<br>My chest feels very tight
当我在爬坡或爬一层楼时，我并不感觉喘不过气来<br>When I walk up a hill or one flight of stairs I am not breathless	0	1	2	3	4	5	当我爬坡或爬一层楼时，我感觉非常喘不过气来<br>When I walk up a hill or one flight of stairs I am very breathless
我在家里的任何劳动都不受慢阻肺的影响<br>I am not limited doing any activities at home	0	1	2	3	4	5	我在家里的任何劳动都很受慢阻肺的影响<br>I am very limited doing activities at home
尽管我有肺病，我还是有信心外出<br>I am confident leaving my home despite my lung condition	0	1	2	3	4	5	因为我有肺病，对于外出我完全没有信心<br>I am not at all confident leaving my home because of my lung condition

调查问卷 v7 2018.10.4
Study questionnaire 041018

我睡得好<br>I sleep soundly	0 1 2 3 4 5	因为我有肺病，我睡得不好<br>I don’t sleep soundly because of my lung condition
我精力旺盛<br>I have lots of energy	0 1 2 3 4 5	我一点精力都没有<br>I have no energy at all

COPD 评估测试和 CAT 的标志是 GlaxoSmithKline 集团公司的商标。
©2009 GlaxoSmithKline 集团公司。版权所有。

17. 您在儿童时期，患过气管炎，肺炎或者严重的百日咳吗？
Did you ever have bronchitis, pneumonia or severe whooping cough as a child?

☐ 有
Yes

☐ 无 如果没有，跳至第 19 题
No If no, ***please go to question 19***

18. 如果有，您患病的时候多大？（或者首次发作的时候）？
If yes, approximately how old were you when you had this (or first time if several episodes)?

___岁
_____years
或
or
_____月
_____months

19. 您在孩童时期，是否患过肺结核？
Did you ever have tuberculosis as a child?

☐ 是
Yes

☐ 否，如果否，请跳至第 21 题
No If no, ***please go to question 21***

20. 如果是，那时您大概多大？（或者第一次患病多大，如果有复发的话）
If yes, approximately how old were you when you had this (or first time if several episodes)?

_____年
_____years
或
Or
______月
______months

调查问卷 v7 2018.10.4
Study questionnaire 041018

21. 以下哪些化学物质或颗粒是您目前正在工作/家中接触的，或者您在工作/家中已经接触过了哪些？(生物质燃料包括木柴，粪肥，农作物残留物如秸秆/草/灌木，煤和煤油)
Which of the following chemicals or particulates are you currently exposed to at work/home, or which have you been exposed to at work/home in the past? (Biomass fuel consists of fire wood, manure, agricultural crop residues such as straw/grass/shrubs, coal fuels and kerosene)

物质种类<br>chemicals or particulates	是，正在接触<br>Yes, currently	是，过去接触过<br>Yes, in the past	否，从没接触过<br>No, never
烹饪油烟<br>Cooking fumes			
生物质燃料<br>Biomass fuel			
各种物质的蒸汽<br>Steam of various substances			
气体<br>Gas			
灰尘<br>Dust			

22. 如果您接触过上述物质，您接触了多少年？
If you ticked ‘yes’ to any exposures, how many years have you been exposed to them?

_______年
______years

23. 如果您接触了烹饪油烟或者生物质燃料，您的家中/工作地点有烟囱或排烟系统吗？
If exposed to cooking fumes or biomass fuels, did the home/workplace have a chimney or exhaust system?

☐ 有
Yes
☐ 无
No

非常感谢您抽出宝贵的时间参与本研究！

Thank you for taking the time to complete this survey

调查问卷 v7 2018.10.4
Study questionnaire 041018
