# Supplementary material for: Study to evaluate the effectiveness and cost-effectiveness of different screening strategies for identifying undiagnosed COPD among residents (≥40 years) in four cities in China: protocol for a multicentre cross-sectional study on behalf of the Breathe Well group
Source: BMJ Open. 2020 Nov 27;10(11):e035738. doi: 10.1136/bmjopen-2019-035738 (PMC7703419; doi:10.1136/bmjopen-2019-035738)
Supplement: Supplementary data [file bmjopen-2019-035738supp003.pdf]

Study ID ☐☐☐☐☐☐

COPD case finding study: assessment of task timing

IMPORTANT: Please write how long each task takes in minutes.

Assessment station 1 – NO TIMING REQUIRED

Assessment station 2

Please only note the time for standing height (not arm span or weight)

Standing height **start time ______ end time ______** ☐☐ minutes

Assessment station 3

Pre-bronchodilator peak flow **start time ______ end time ______** ☐☐ minutes

Pre-bronchodilator microspirometry **start time ______ end time ______** ☐☐ minutes

Assessment station 4

Administration of Salbutamol **start time ______ end time ______** ☐☐ minutes

Assessment station 5

Completion of Lung Health questionnaire (CDQ etc) **start time ______ end time ______** ☐☐ minutes

Did the patient require assistance? Yes ☐ No ☐

If yes, was assistance required for the whole questionnaire? Yes ☐ No ☐

Assessment station 6

Post-bronchodilator spirometry **start time ______ end time ______** ☐☐ minutes
