# Supplementary material for: Study to evaluate the effectiveness and cost-effectiveness of different screening strategies for identifying undiagnosed COPD among residents (≥40 years) in four cities in China: protocol for a multicentre cross-sectional study on behalf of the Breathe Well group
Source: BMJ Open. 2020 Nov 27;10(11):e035738. doi: 10.1136/bmjopen-2019-035738 (PMC7703419; doi:10.1136/bmjopen-2019-035738)
Supplement: Supplementary data [file bmjopen-2019-035738supp004.pdf]

# 北京大学第一医院生物医学研究伦理委员会审查批件

伦理审查编号：（2018）科研第（141）号-修正案　　EC 存档档案号：2018 研 141

伦理委员会批准日期：2018 年 11 月 07 日　批件有效期至：2019 年 11 月 06 日　定期跟踪审查频率：12 个月

| | | | |
|---|---|---|---|
| 项目名称 | 慢性阻塞性肺疾病不同筛查策略在中国四个城市≥40 岁人群中的有效性及成本-效益的评价性研究-横断面研究 | | |
| CFDA 批件号 | / | | |
| 申办者 | 北京大学第一医院　健康管理中心 | | |
| 临床研究科室 | 健康管理中心 | 主要研究者 | 迟春花 |
| 批准的文件 | 1、研究方案（版本号：6　版本日期：2018.10.25）<br>2、知情同意书（版本号：6.0　版本日期：2018.10.25）<br>3、研究对象信息表（版本号:v1　版本日期：2018.10.19）<br>4、肺部健康问卷（版本号：1.0　版本日期：2018.5.9）<br>5、调查问卷（版本号：v7　版本日期：2018.10.4）<br>6、中国案例报告表（版本号：Version3　版本日期：2018.10.25） | | |

**本伦理委员会的人员组成和工作程序符合中国 GCP 以及国家相关规定**

伦理审查方式：■快速审查　审查时间：2018 年 11 月 07 日

| | |
|---|---|
| 审查委员 | 张宝娓　谢鹏雁 |
| 审查意见 | 同意按照上述批准的文件进行该临床试验。 |

注意事项：

1. 本项临床试验应当在伦理委员会同意进行之日起 1 年内实施。逾期未实施的，本审查批件自行废止。
2. 研究应遵循本伦理委员会批准的方案执行，须符合 GCP 和《赫尔辛基宣言》的原则。
3. 自同意研究之日起，每隔 12 个月伦理委员会的定期跟踪审查（审查频度可能根据实际进展情况改变）；请在定期跟踪审查到期前 1 个月递交《定期跟踪审查表》。
4. 研究过程中，对研究方案和知情同意书等相关文件所作的任何修改，请交《修正案申请表》及“送审文件清单”中规定相关资料，并得到伦理委员会审查同意该修正后方可实施。
5. 发生严重不良事件或影响研究风险受益比的非预期不良事件，在向 CFDA 上报的同时向伦理委员会作书面通报，可以使用 CFDA 的《严重不良事件报告表》或本伦理委员会公布的《严重不良事件/非预期不良事件报告表》或其他有相关内容的报告表，但外文的报告需要有中文摘要。伦理委员会有权根据对其评估做出新的决定。
6. 不依从或违反方案应及时提交《不依从或违反方案报告表》。
7. 提前终止研究应及时提交《研究方案提前终止报告表》。
8. 研究完成后提交《研究结题报告表》和临床试验总结报告。
9. 及时书面报告其他伦理委员会的重要决定。

主任委员或副主任委员签名：

北京大学第一医院生物医学研究伦理委员会（盖章）

2018年　11 月　7 日

伦理委员会地址：北京市西城区大红罗厂街 6 号　邮编：100034　联系电话：010-66119025
