# Supplementary material for: Study to evaluate the effectiveness and cost-effectiveness of different screening strategies for identifying undiagnosed COPD among residents (≥40 years) in four cities in China: protocol for a multicentre cross-sectional study on behalf of the Breathe Well group
Source: BMJ Open. 2020 Nov 27;10(11):e035738. doi: 10.1136/bmjopen-2019-035738 (PMC7703419; doi:10.1136/bmjopen-2019-035738)
Supplement: Supplementary data [file bmjopen-2019-035738supp006.pdf]

Evaluating screening strategies for identifying undiagnosed COPD in China: a Breathe Well project

Informed consent

We are conducting a study to evaluate the accuracy of different screening methods for chronic obstructive pulmonary disease (COPD) in people aged 40 years and older. We are inviting you to participate in this study because your condition meets the criteria for the enrolment. This sheet will give you a brief introduction for the purpose, process, benefits, and risks of this study. Please read it carefully before deciding whether you are interested in taking part, and you are welcome to discuss it with your family and friends. When the researcher explains and discusses the informed consent form, you can ask questions and ask him/her to explain anything to you if you have anything you don't understand. You can make a decision after discussing with your family, friends, and your doctor.

The study is funded through a collaboration with the University of Birmingham in the UK The primary investigator of this study is Chi Chunhua, chief physician of Peking University First Hospital.

1. Why do we conduct this study?

COPD has brought a huge economic burden to the country and has also brought about psychological and economic hardships for patients. China has a large population of patients with COPD. However, it faces the problems of high rate of missed and late diagnosis. The community is an important checkpoint for the prevention and treatment of chronic non-communicable diseases, and the community has unique advantages in early detection and early intervention in the COPD population. However, at present, what is cost-effective screening method for early detection of COPD patients is not clear. In view of this, we designed this study to assess the best screening methods for COPD in the community in China. The test commonly used to assess people's lung health is called spirometry, and this will be compared against screening tests including a peak flow meter, microspirometry and questionnaires.

2. Who will be invited to participate in this study?

Eligibility criteria:

Inclusion

- Aged ≥40 years
- Residing in the catchment areas

Exclusion

- Unable to do spirometry (e.g. dementia, lack of teeth-cannot make a good seal)
- Contraindicated for spirometry (chest infection in the last 3 weeks, coughing up blood in the last month, severe angina, uncontrolled high blood pressure, pneumothorax or history in the last 3 months of tuberculosis, heart attack, detached retina, or surgery on chest/abdomen/brain/ears/eyes
- Currently pregnant/breastfeeding
- Previous adverse reaction to Salbutamol

In addition, the researcher will judge according to your actual situation whether you are suitable for this study.

3. How many people will be recruited in this study?

We are recruiting approximately 2,000 subjects in the study, each community health centres need to recruit nearly 250 subjects.

4. What is the process of the study?

This study is a multi-centre cross-sectional survey (diagnostic test accuracy study). The research sites are Beijing (North), Chengdu (Southwest), Guangzhou (South), and Shenyang (Northeast). Each city selects two research sites: a community health service centre in urban area, and another in rural area.

Instruments model and drug information:

Instruments:

1） Expiratory peak flow meter(PEF): USPE

2） Microspirometer: COPD-6

3) Spirometer: ndd Easy On-PC (ndd Medizintechnik AG)

Drug:

Bronchodilator: Salbutamol Aerosol (GSK)

The instruments and drugs used in this study have been approved by the State Food and Drug Administration (CFDA).

Study assessment visit:

If you participate in the study, you would attend one study assessment visit lasting approximately 90 minutes in total. If you are eligible for the study you will have your height and weight measured, before being asked to do three blowing tests and complete study questionnaires.

The data collection process is as flows:

Assessment station 1:

The researcher will confirm your eligibility for the study based on the criteria mentioned above and check for current chest infection (those with acute infections will be rebooked). If you are eligible you will then have opportunity to ask any questions about the study before completing the consent form with the researcher. As well as consenting to the main study, you will be asked to consent to being contacted about future research related to the study. Future contact will be optional and will not affect your ability to take part in the study.

Assessment station 2:

The researcher will measure your height and weight.

Assessment station 3:

You will be asked to perform blowing tests on two different devices, one called a peak flow meter and one called a microspirometer. You will be asked to perform 3 blows on each device, and further details are given below.

Lung function (peak flow)

A trained researcher will assess peak expiratory flow using a simple peak flow meter (USPE). For the main analysis, peak expiratory flow rates (PEFR) of <350 l/min for men and <250 l/min for women will be used to indicate a positive test Standardized researcher training will detail how to perform the technique and how to use the device. Each participant will perform three blows without administration of salbutamol, after which the researcher will record the highest PEF.

Lung function (microspirometry)

Microspirometry will be performed with minimal coaching by a trained researcher using a simple handheld microspirometer (COPD6), to measure FEV_1, FEV_6 and FEV_1/FEV_6 ratio. Microspirometer devices will be checked for calibration errors at the start of the study by the researchers. Standardized researcher training will detail how to perform the technique and how to use the device. Each participant will perform three blows using the device, after which the researcher will record the highest FEV_1 and FEV_6 values and the FEV_1/FEV_6 ratio. For the main analysis, FEV1/FEV6 ratios of <0.75 and <0.78 will be used to indicate a positive test.

Assessment station 4:

You will receive 400 µg of salbutamol using a large volume spacer, and then wait approximately 20 minutes before performing another blowing test.

Assessment station 5:

During the 20 minutes waiting period, you will be asked to complete study questionnaires by yourself, in a separate area of the assessment clinic, and return the completed forms to the researcher. The study pack will include four screening questionnaires (CAPTURE, CDQ, COPD-SQ and a symptom-based questionnaire). In addition to the screening questionnaires, the study pack will also include items relating to the following topics: demographic data (sex, age, marital status, education level, deprivation); lifestyle (smoking status, exercise); exposures (biomass smoke,

occupational exposure to chemicals and particulates); health (medical diagnoses, comorbidities, respiratory symptoms, use of medications); quality of life (COPD assessment test (CAT)).

A member of the research team will be available to help you complete questionnaires if necessary.

Assessment station 6:

After completing the questionnaires, you will be asked to perform another blowing test using a portable spirometer (ndd Easy On-PC) that will be linked to a laptop. You may be asked to repeat this blowing test up to a maximum of 6 times.

5. How long will this study last?

This study will last for approximately one year.

6. What are the risks of participating in this study?

Blowing tests will be performed multiple times depending on the research needs. Multiple examinations may cause discomfort to some patients. If any discomfort occurs, please inform the researchers at any time. We will give you Salbutamol before the final blowing test. There is a very small risk of drug allergy. If you have adverse reactions with salbutamol in this study, please be sure to inform the investigator. Possible side effects from Salbutamol are rare and include feeling shaky, rapid heart rate or headache. If you experience any of these, it will be immediately after taking Salbutamol and will disappear after several minutes

Some questions in the questionnaire may make you feel uncomfortable, if so, ,you can refuse to answer.

7. What are the benefits of participating in this study?

You will not benefit directly from participating in this study. Your participation will help us find an effective, cost-effective method of screening for COPD, so that patients with COPD can be detected early, and they can be treated promptly to relieve their condition.

Funded by NIHR

At the end of the study assessment, researchers will explain your lung function results to you and answer any immediate questions you may have.

8. Is it necessary to participate in and complete this study?

It is entirely voluntary whether you participate in this research. If you do not want to, you can refuse to participate, which will not have any negative impact on your current or future medical care. Even if you agree to participate, you can change your mind at any time and tell the researchers to withdraw from the study. Your withdrawal will not affect your access to normal medical services.

If you decide to withdraw from this study, we will stop collecting new data related to this study from you.

9. Fees and compensation for participating in the study

You don't need to pay any cost to participate in this study.

We will compensate you for your time and support for participation by giving you small household tokens (e.g. washing powder, toothpaste, liquid soap).

This study does not provide monetary compensation such as transportation fees and losing of working time.

10. What happened to research-related injuries?

There are no relevant invasive tests in this study.

If you have any discomfort during the research process, please inform the researchers. We will promptly take the necessary medical measures for treatment or inform the researcher (main researcher **Pan Zihan**, Tel. **18701291196**). If it is confirmed that the health status is harmed because of participating in this study, you'll be compensated according to the current laws.

11. Is my information confidential?

If you decide to participate in this study, your participation in the study and personal data are confidential. Information that identifies you will not be disclosed to anyone other than the research member unless we have your permission. All research members are required to keep your identity confidential. Your file will be kept in a

locked file cabinet for research purposes only. In order to ensure that the research is conducted in accordance with the regulations, members of the government management department or ethics committee may, as required, consult your personal data in the research sites.

12. Data storage

Data will be stored securely at the community centre, the co-ordinating study centre and on an online database that is held on servers at the University of Birmingham, UK. For further information, please refer to the Breathe Well website (https://www.birmingham.ac.uk/breathewell).

13. Publication of study findings

When we have analysed the results we will publish them in an academic journal. All publications will be available on the Breathe Well website. You will not be identified in any publication.

14. Who should I contact if I have problems or difficulties?

If you have any problems or difficulties related to this study, please contact **Dr. Pan Zihan**, her telephone number is **18701291196**.

If you have any problems or difficulties related to the subject's own rights, you can contact the Biomedical Research Ethics Committee of the **Peking University First Hospital ,**Tel: **010-66119025**.

15. Funding

The research was commissioned by the National Institute for Health Research using Official Development Assistance (ODA) funding. The views expressed are those of the author(s) and not necessarily those of the NHS, the NIHR or the Department of Health.

BUILDING RESEARCH ACROSS THE WORLD IN LUNG DISEASE

16. Signature

Study ID: __________________

Subject statement

The investigators explained to me the background, purpose, process, risks, and benefits of participating in ”a study to evaluate the accuracy of different screening methods for chronic obstructive pulmonary disease（COPD）in people aged 40 years and older”. I have enough time and opportunity to ask questions. I am very satisfied with the answers provided by the researchers. I know who I should contact when I have questions or want to get further information. I read this informed consent and decided to participate in this study. I know that I can withdraw from this study at any time during the study without any reason. I was told I would get a copy of this informed consent, which contains the signature of me and the researcher.

I give my permission to be contacted in the future for related research purposes including other studies in the Breathe Well programme. ☐

Subject's signature: Date:

Legal representative's signature [if applicable]: Date:

Relationship with the subject:

Researcher's statement

I have explained to the subjects (and legal representatives) the background, purpose, process, risks, and benefits of participating in “a study to evaluate the accuracy of different screening methods for chronic obstructive pulmonary disease（COPD）in people aged 40 years and older”. He/she had enough time to read the informed consent form, discuss it with others, and we answered his/her questions about the study; I told the subject how to contact the persons when they have research-related questions; I told him/her (or legal representative) that he/she may withdraw this study without any reason at any time during the study.

Researcher's signature: Date:

Funded by NIHR
