# Supplementary material for: Study to evaluate the effectiveness and cost-effectiveness of different screening strategies for identifying undiagnosed COPD among residents (≥40 years) in four cities in China: protocol for a multicentre cross-sectional study on behalf of the Breathe Well group
Source: BMJ Open. 2020 Nov 27;10(11):e035738. doi: 10.1136/bmjopen-2019-035738 (PMC7703419; doi:10.1136/bmjopen-2019-035738)
Supplement: Supplementary data [file bmjopen-2019-035738supp005.pdf]

**发件人:**	Susan Cottam <s.l.cottam@bham.ac.uk>
**发送时间:**	2018年10月22日星期一 17:30
**收件人:**	Andy Dickens
**主题:**	Application for Ethical Review ERN_18-1177

Dear Dr Dickens

Re: "A study to evaluate the effectiveness and cost-effectiveness of different screening strategies for identifying undiagnosed COPD in China, amongst residents (≥ 40 years) in four cities"
Application for Ethical Review ERN_18-1177

Thank you for your application for ethical review for the above project, which was reviewed by the Science, Technology, Engineering and Mathematics Ethical Review Committee.

On behalf of the Committee, I confirm that this study now has full ethical approval.

I would like to remind you that any substantive changes to the nature of the study as described in the Application for Ethical Review, and/or any adverse events occurring during the study should be promptly bought to the Committee's attention by the Principal Investigator and may necessitate further ethical review.

Please also ensure that the relevant requirements within the University's Code of Practice for Research and the information and guidance provided on the University's ethics webpages (available at https://intranet.birmingham.ac.uk/finance/accounting/Research-Support-Group/Research-Ethics/Links-and-Resources.aspx) are adhered to and referred to in any future applications for ethical review. It is now a requirement on the revised application form (https://intranet.birmingham.ac.uk/finance/accounting/Research-Support-Group/Research-Ethics/Ethical-Review-Forms.aspx) to confirm that this guidance has been consulted and is understood, and that it has been taken into account when completing your application for ethical review.

Please be aware that whilst Health and Safety (H&S) issues may be considered during the ethical review process, you are still required to follow the University's guidance on H&S and to ensure that H&S risk assessments have been carried out as appropriate. For further information about this, please contact your School H&S representative or the University's H&S Unit at healthandsafety@contacts.bham.ac.uk.

Kind regards

Susan Cottam
Research Ethics Officer
Research Support Group
C Block Dome
Aston Webb Building
University of Birmingham
Edgbaston B15 2TT
Tel: 0121 414 8825
Email: s.l.cottam@bham.ac.uk
Web: https://intranet.birmingham.ac.uk/finance/RSS/Research-Support-Group/Research-Ethics/index.aspx

Please remember to submit a new Self-Assessment Form for each new project.

You can also email our team mailbox ethics-queries@contacts.bham.ac.uk with any queries relating to the University's ethics process.

Click Research Governance for further details regarding the University's Research Governance and Clinical Trials Insurance processes, or email researchgovernance@contacts.bham.ac.uk with any queries relating to research governance.

Notice of Confidentiality:
The contents of this email may be privileged and are confidential. It may not be disclosed to or used by anyone other than the addressee, nor copied in any way. If received in error please notify the sender and then delete it from your system. Should you communicate with me by email, you consent to the University of Birmingham monitoring and reading any such correspondence.

Pan Z, *et al. BMJ Open* 2020; 10:e035738. doi: 10.1136/bmjopen-2019-035738
