# Supplementary material for: Study to evaluate the effectiveness and cost-effectiveness of different screening strategies for identifying undiagnosed COPD among residents (≥40 years) in four cities in China: protocol for a multicentre cross-sectional study on behalf of the Breathe Well group
Source: BMJ Open. 2020 Nov 27;10(11):e035738. doi: 10.1136/bmjopen-2019-035738 (PMC7703419; doi:10.1136/bmjopen-2019-035738)
Supplement: Author's manuscript [file bmjopen-2019-035738.draft_revisions.pdf]

BMJ Open

BMJ Open is committed to open peer review. As part of this commitment we make the peer review history of every article we publish publicly available.

When an article is published we post the peer reviewers' comments and the authors' responses online. We also post the versions of the paper that were used during peer review. These are the versions that the peer review comments apply to.

The versions of the paper that follow are the versions that were submitted during the peer review process. They are not the versions of record or the final published versions. They should not be cited or distributed as the published version of this manuscript.

BMJ Open is an open access journal and the full, final, typeset and author-corrected version of record of the manuscript is available on our site with no access controls, subscription charges or pay-per-view fees (http://bmjopen.bmj.com).

If you have any questions on BMJ Open's open peer review process please email info.bmjopen@bmj.com

BMJ Open

A study to evaluate the effectiveness and cost-effectiveness of different screening strategies for identifying undiagnosed COPD amongst residents (≥40 years) in four cities in China: protocol for a multicenter cross-sectional study. On behalf of the Breathe Well group.

Journal:	*BMJ Open*
Manuscript ID	bmjopen-2019-035738
Article Type:	Protocol
Date Submitted by the Author:	14-Nov-2019

Complete List of Authors:	Pan, Zihan; Peking University First Hospital, ; Peking University Health Science Centre,<br>Dickens, Andrew; University of Birmingham, Institute of Applied Health Research<br>Chi, chunhua; Peking University First Hospital, General Practice<br>Kong, Xia; Peking University First Hospital, General Practice<br>Enocson, Alexandra; University of Birmingham, Applied Health Research<br>Adab, Peymane; University of Birmingham Institute of Applied Health Research, Institute of Applied Health Research<br>Cheng, Kar Keung; University of Birmingham Institute of Applied Health Research<br>Sitch, Alice; University of Birmingham Institute of Applied Health Research<br>Jowett, Sue; University of Birmingham Institute of Applied Health Research<br>Jordan, Rachel; University of Birmingham Institute of Applied Health Research

Keywords:	Chronic airways disease < THORACIC MEDICINE, HEALTH ECONOMICS, PUBLIC HEALTH, PRIMARY CARE

SCHOLARONE™
Manuscripts

I, the Submitting Author has the right to grant and does grant on behalf of all authors of the Work (as defined in the below author licence), an exclusive licence and/or a non-exclusive licence for contributions from authors who are: i) UK Crown employees; ii) where BMJ has agreed a CC-BY licence shall apply, and/or iii) in accordance with the terms applicable for US Federal Government officers or employees acting as part of their official duties; on a worldwide, perpetual, irrevocable, royalty-free basis to BMJ Publishing Group Ltd ("BMJ") its licensees and where the relevant Journal is co-owned by BMJ to the co-owners of the Journal, to publish the Work in this journal and any other BMJ products and to exploit all rights, as set out in our licence.

The Submitting Author accepts and understands that any supply made under these terms is made by BMJ to the Submitting Author unless you are acting as an employee on behalf of your employer or a postgraduate student of an affiliated institution which is paying any applicable article publishing charge ("APC") for Open Access articles. Where the Submitting Author wishes to make the Work available on an Open Access basis (and intends to pay the relevant APC), the terms of reuse of such Open Access shall be governed by a Creative Commons licence – details of these licences and which Creative Commons licence will apply to this Work are set out in our licence referred to above.

Other than as permitted in any relevant BMJ Author's Self Archiving Policies, I confirm this Work has not been accepted for publication elsewhere, is not being considered for publication elsewhere and does not duplicate material already published. I confirm all authors consent to publication of this Work and authorise the granting of this licence.

For peer review only

A study to evaluate the effectiveness and cost-effectiveness of different screening strategies for identifying undiagnosed COPD amongst residents (≥40 years) in four cities in China: protocol for a multicenter cross-sectional study. On behalf of the Breathe Well group.

Zihan Pan[1]; Andrew P Dickens[2]; *Chunhua Chi[1]; Xia Kong[1]; Alexandra Enocson[2]; Peymané Adab[2]; Kar Keung Cheng[2]; Alice Sitch[2,3]; Sue Jowett[2], Rachel E Jordan[2] on behalf of the Breathe Well Group.

Corresponding author:
Name: Chunhua Chi
Postal address: Peking University First Hospital, No.8 XiShiKu Street, Xicheng District, Beijing, 100034,China
E-mail: chichunhua@vip.163.com,
Telephone: +86 13910987530,
Fax numbers: +86 010-66158996

Co-authors details:
1. Department of General Practice, Peking University First Hospital, Beijing, China
2. Institute of Applied Health Research, University of Birmingham, Birmingham, UK.
3. NIHR Birmingham Biomedical Research Centre, University Hospitals Birmingham NHS Foundation Trust and University of Birmingham, UK

Key word: COPD; diagnostic accuracy test; screening strategies, health economics; primary care

Word count: 3952 words

For peer review only

Abstract

Introduction: The latest COPD epidemiology survey in China estimated that there were 99 million potential COPD patients in the country, the majority of whom are undiagnosed. Screening for COPD in primary care settings is of vital importance for China, but it is not known which strategy would be the most suitable for adoption in primary care. Studies have been conducted to test the accuracy of questionnaires, expiratory peak flow meters, and microspirometers to screen for COPD, but no studies have directly evaluated and compared the effectiveness and cost-effectiveness of these methods in the Chinese setting.

Methods and analysis: We present the protocol for a multicenter cross-sectional study, to be conducted in 8 community hospitals from 4 cities amongst Chinese adults aged 40 years or older to investigate the effectiveness and cost-effectiveness of different case finding methods for COPD, and determine the test performance of individual and combinations of screening tests and strategies in comparison with quality diagnostic spirometry. Index tests are screening questionnaires (CDQ, CAPTURE, symptom-based questionnaire, COPD-SQ), microspirometer and peak flow. The reference test is quality diagnostic spirometry. Each participant will complete all of these tests in one assessment. Approximately 2000 participants will be recruited over 9-12 months.

Ethics and dissemination: The study has been approved by Peking University Hospital and University of Birmingham. All study participants will provide written informed consent. Study results will be published in appropriate journal and presented at national and international conferences, as well as relative social media and various community/stakeholder engagement activities.

Trial registration: ISRCTN13357135.

Keywords: COPD; diagnostic accuracy test; screening strategies, health economics; primary care

For peer review only

Strengths and limitations of the study

- The first study to compare the test performance of selected screening tests and strategies to screen for COPD in China, including questionnaires, peak flow meter and microspirometer.
- Collected data will identify the most effective and cost-effective COPD screening strategies in primary care settings in China, and provide a reference for other similar settings.
- Blinded administration of quality diagnostic spirometry minimises risk of review bias.
- The study will be conducted in 4 cities across China, which are geographically disparate but may not be totally representative of China as a whole.

Introduction

Chronic obstructive pulmonary disease (COPD) is a common, preventable and treatable chronic condition characterized by persistent respiratory symptoms and air flow limitation [1]. Although it is well known that COPD has been the 3rd leading cause of death in 2010 in the world [2], COPD is underdiagnosed throughout the world because of the low awareness of the general population and its consequences, the low awareness of the doctors in charge, and the low use of spirometry[3]. 73% of individuals with non-reversible airflow obstruction compatible with COPD were not diagnosed in the Spanish EPI-SCAN study [3], 71.4% of COPD patients had not been diagnosed with COPD in the study from Poland[4]. However, underdiagnosed COPD is more critical in China. As shown in the recent national COPD epidemiology study in China, only 4% of COPD patients identified by spirometry had previously been diagnosed with COPD[5]. Subjects with undiagnosed COPD were characterized by fewer symptoms[6]. The underdiagnosed COPD contained 68% of asymptomatic people in China[7], what's more, about 30%COPD patients were asymptomatic, those people were more likely to be underdiagnosed[4, 8].

Although the US Preventive Services Task Force (USPSTF) recommends against screening for COPD in asymptomatic adults because of scant evidence showing a benefit of early detection and treatment, studies suggested that for the population with at least one risk factor or respiratory symptom, screening by spirometry has changed management and outcomes related to COPD[9]. The Global Initiative for Chronic Obstructive Lung Disease(GOLD) denied subjects who met at least one of the following conditions, including chronic respiratory symptoms, exposure to risk factors, medical history of respiratory diseases, and family history as population at high risk[1]. According to the above definition, about 90% of people aged ≥40 years in China were at high risk of COPD in 2014[10], moreover, the prevalence of COPD was 13.7%(in 2015)[5]. Faced with high underdiagnoses, high prevalence and high proportion of risk population, it is essential and imperative to screen for COPD in China. Recently, China has called for national policy and programmes for the prevention and early detection of COPD[5,11]. Fortunately, government agencies have recommended the incorporation of pulmonary function tests into routine health examinations in China's 13th Five-Year Plan for Health care[12].

Spirometry is recommended as a diagnostic tool in GOLD[1]. For a variety of reasons, however, spirometry is not always available in primary care settings in China [13, 14]. Among a large population of COPD patients in China, only 5.9-12% had ever been tested using spirometry[5, 11]. Simple and affordable tools and methods are needed for COPD screening in primary care settings where

For peer review only

spirometry is unavailable.

Even though national policies do recommend screening for undiagnosed COPD in China, but there is no recommendations on the best strategy or approach for COPD screening. Many researches internationally had examined the effectiveness of various screening tests for COPD, such as questionnaires and simple lung function tests. Multiple screening questionnaires have been developed to identify patients at risk of COPD, either in primary or secondary care settings [15-19]. Questionnaire items include the presence of respiratory symptoms (e.g. wheeze, dyspnea, cough) while some tools also explore topics such as exposures, smoking history and age. The questionnaires are all designed to be self-completed, but vary regarding the populations in which they were developed/validated e.g. general population or targeted groups such as symptomatic patients, current smokers etc. Microspirometers are small handheld devices that measure lung function, which are low cost, require minimal coaching for patients and are quick to use. Evidence suggests that they are suitable for use as a screening tool to identify patients who merit referral for quality diagnostic spirometry[20-24]. Peak flow monitors are simple, low cost devices that measure how much air patients can expel during a forced expiration (peak expiratory flow, PEF). Peak flow tests can therefore be used to assess respiratory conditions, and recent studies have explored their used as a possible screening tool for COPD[17,25, 26].

Screening tests can be used in isolation or in combination as screening 'strategies'. Although some studies compared the screening accuracy between different questionnaires[27-30], the comparison between screening questionnaires and microspirometers or peak flow monitors or different combinations was not enough. What's more, studies in community settings in China are limited and it is not known which strategy would be the most suitable for adoption in this settings. As a middle-income country with a huge potential COPD population, it would be beneficial to explore the effective and cost-effective screening strategies in China that could identify patients who merit referral to quality diagnostic spirometry. Accurately detecting these individuals could minimize the number of ineligible referrals thus protecting health system resources, and ensure those subsequently diagnosed receive treatment in a timely manner.

Methods and analysis

Study recruitment commenced in February 2019 and is anticipated to continue for approximately 9-12 months.

Aims and objectives

Aim

The aim of the study is to identify the most effective and cost effective screening strategy for identifying undiagnosed COPD amongst those aged 40 years or older in China.

Objectives

- To determine the comparative test performance of all screening tests and strategies in diagnosing COPD (confirmed by quality diagnostic spirometry).
- To evaluate the cost-effectiveness of each screening strategy.

Design

Multicenter cross-sectional test accuracy study. The study is registered at *http://www.isrctn.com*

For peer review only

(ISRCTN13357135).

The STARD guideline[31] was used for reporting studies of diagnostic test accuracy to inform the content of the protocol and we will use this to report the study.

Study setting

The study will be implemented in four cities in China: Beijing (North), Chengdu (Southwest), Guangzhou (South), Shenyang (Northeast). Cities were purposively selected to represent urban/rural settings and differing geographic areas of the country, where exposures, lifestyles and the prevalence of COPD may differ. The national study about COPD prevalence in 2007 in China was taken as a selection reference. Each selected city had the highest prevalence of COPD in each geographic area, prevalence is shown on the map[32]. Participants will be recruited from eight community health service centers (CHSC); 1 rural and 1 urban in each city. The study sites are shown on the map (Figure 1).

Study population

Inclusion criteria

- Aged ≥40 years
- Residing in the catchment areas of the participating CHSCs in the four cities

Exclusion criteria

- Unable to perform spirometry (e.g. dementia, lack of teeth-cannot make a good seal)
- Contraindicated for spirometry (chest infection in the last 3 weeks, coughing up blood in the last month, severe angina, uncontrolled high blood pressure, pneumothorax or history in the last 3 months of tuberculosis, heart attack, detached retina, or surgery on chest/abdomen/brain/ears/eyes
- Currently pregnant/breastfeeding
- Previous adverse reaction to Salbutamol

Recruitment

Participants will be recruited to the study via two main routes; advertisement or doctor referral. Participating CHSCs and their satellite offices will advertise the study by displaying posters and sending messages to their secure/closed/other resident WeChat social media groups, inviting residents to contact the research team if they are interested in taking part. Potentially eligible patients visiting the participating CHSCs will be given a study information sheet by the healthcare professionals and invited to attend a study assessment with researchers. Study participants will also be encouraged to promote the study to their family members and friends. The recruitment route of all participants will be recorded.

For the first 4 weeks of recruitment, the study will only be conducted in Beijing to allow all study processes to be piloted and altered as required, after which it will be implemented in the other 3 cities. Recruitment flow through the study is summarized in Figure 2.

Study tests

For peer review only

The study will use a paired design, with all participants receiving the index tests and reference test during the same study assessment. The study will administer a total of 6 index tests (pre-bronchodilator peak flow and micro spirometry, and 4 screening questionnaires) and one reference test (post-bronchodilator quality diagnostic spirometry) to each participant.

Index tests

- Lung function test—Peak flow

A trained researcher will assess peak expiratory flow using a simple peak flow meter (USPE, China). Each participant will perform three blows without administration of bronchodilator, after which the researcher will record the highest PEF. For the main analysis, peak expiratory flow rates (PEFR) of <350 l/min for men and <250 l/min for women will be used to indicate a positive test[17].

- Lung function test—Microspirometer

Micro spirometry will be performed with minimal coaching by a trained researcher using a simple handheld microspirometer (Vitalograph COPD6), to measure FEV_1, FEV_6 and FEV_1/FEV_6 ratio. Each participant will perform three blows using the device, after which the researcher will record the highest FEV_1 and FEV_6 values and the FEV_1/FEV_6 ratio. For the main analysis, FEV_1/FEV_6 ratios of <0.75[33] and <0.78[24] will be assessed to indicate a positive test.

- Screening questionnaires

Four screening questionnaires will be used in the study; the CDQ[16,34], the COPD-SQ[18], a symptom-based questionnaire[35], and CAPTURE[17](see Appendix 1). The selection of questionnaires maximizes symptoms being assessed and minimizes duplication of items, whilst allowing comparison of the most relevant questionnaires. Recommended cut-points for each questionnaire will be used to identify those at risk of COPD for diagnostic spirometry, with potential additional analyses to explore optimal cut-points.

Reference test

Post-bronchodilator quality diagnostic spirometry (20-60 minutes after administration of 400ug Salbutamol) will be performed by a trained researcher using a portable spirometer (ndd Easy On-PC). Lung function data including FEV_1, FVC and FEV_1/FVC ratio will be recorded in the ndd software, and will also be imported to the study REDCap database. Accuracy of the device flow heads will be verified at the start of each assessment day by the researchers; calibration is not required. Participants will perform a maximum of six blows, or less if repeatability within 100mls or 5% is achieved (ARTP standards (2013))[36]. For the purposes of this study, a COPD diagnosis will be defined as airflow obstruction based on the lower limit of normal using the Global Lung Initiative (GLI) equations, according to post-bronchodilator quality diagnostic spirometry.

Ordering of assessments

Index tests will be conducted before the reference test for all participants, and the reference test will be administered by a different researcher who will be blind to the previous test results. To decrease the potential training effect within the index tests, the order of the peak flow and microspirometer will be alternated i.e. approximately half of the participants will perform peak flow first and vice versa.

Besides that, participants' standing height (stadiometer) and weight (scales) will be measured. Participants will also be asked to complete a study questionnaire by themselves, in a separate area

For peer review only

of the assessment clinic, and return the completed forms to the researcher. The study questionnaire will include items relating to the following topics: demographic data (sex, age, marital status, education level, deprivation); smoking status; exposures (biomass smoke, occupational exposure to chemicals and particulates); health (medical diagnoses [inc. COPD, asthma, TB etc], comorbidities, respiratory symptoms); quality of life (COPD assessment test (CAT)). A member of the research team will be available to help participants to complete questionnaires if necessary.

Data collection

Study assessment

The study assessment will last approximately 80 minutes, including 6 stations. There will be 2 researchers at each assessment clinic to enable the assessments to run in parallel, and all data will be recorded on CRFs(case report form), ensuring standardized data collection/recording.

At the end of the study assessment, researchers will provide all participants with information about the level of their airway obstruction, suggest they contact a doctor if appropriate, and answer any immediate questions they may have. The flow of participants is presented in figure 2.

Resource use data

To calculate the health care costs of delivering each screening strategy, we will determine the unit costs and quantity of any equipment, medication and consumables required, as well as staff type and grade, staff time taken to deliver each individual test and use of facilities. Equipment costs (peak flow meters, spirometers) will be amortised over the estimated lifespan of the equipment. The cost per patient visit will be calculated using assumptions regarding the total number of patients the equipment will be used for. In addition, each individual test will be timed at a sample of assessment clinics so an overall mean time and range for each test can be estimated.

Statistical methods

Sample size

The Alonzo method for paired test accuracy studies[37] was used to calculate the sample size, assuming independence of tests and a prevalence of 12%, we will have 90% power to detect a difference in sensitivity of 10% (95% vs 85%[17,20,22,34]) with 1622 participants. If the sensitivity of tests is slightly lower in this population (90% vs 80%) we would have 90% power to detect this difference with a larger sample of 2279 participants.

Analysis plan

Data will be analysed using Stata v15.

Our primary analysis will compare the performance of a screening questionnaire (CAPTURE) with a handheld device (peak flow meter). Secondary analyses will include the comparative performance of each index test, as well as a comparison of strategies where we use a screening questionnaire and a handheld device.

The performance of each index test when diagnosing COPD (confirmed by quality diagnostic spirometry) will be investigated by presenting 2x2 tables and calculating the sensitivity, specificity, positive predictive value and negative predictive value, along with 95% confidence intervals. For the tests with a continuous score, receiver operator curve (ROC) analysis with AUC (with 95% CIs)

For peer review only

will be produced. Comparisons of test accuracy between different index tests and different test strategies will be conducted using McNemar's test and logistic regression modelling.

Sensitivity analyses will explore the impact on test performance of the index tests and strategies when using different definitions of COPD, including i) a combination of spirometry data and clinical confirmation, and ii) using the GOLD definition of airflow obstruction. Additional sensitivity analyses may explore optimal cut-points for the screening tests.

A fully incremental cost-effectiveness analysis will be undertaken from a health care perspective to calculate the cost per true case detected for all pre-determined strategies. The strategies (including combinations) will be ordered by the number of true cases detected, from least to greatest, and the principles of dominance and extended dominance will be applied to eliminate redundant strategies from the analysis. Sensitivity analysis will be undertaken to explore the impact on results of any changes in assumptions, e.g. time taken for a strategy.

Data storage

Study data will be entered into a bespoke REDCap online database. All electronic data held by the research team will be password protected and stored on encrypted study laptops. Paper-based data will be held in locked filing cabinets in the study office in each site. The research team will conduct monitoring visits of all research sites during the recruitment period to ensure data are being collected, entered and stored according to pre-specified study working instructions.

Training

A two-day training event will be organized for all researchers to ensure standardized study processes are followed at all research sites. Training will cover study processes and assessment techniques as well as expert teaching regarding respiratory physiology and spirometry lung function tests. Researchers' competency in conducting spirometry will be certified at the end of the training. Spirometry traces from practice sessions will be over-read by an expert to ensure sufficient quality prior to participant recruitment commences. Local respiratory specialists will over-read all spirometry tests during the study period, to ensure quality is maintained. During site initiation visits, the study team will observe a complete study assessment to ensure researchers adhere to the study protocol. The study will conduct monitoring site visits throughout the study period.

Ethics and dissemination

Ethics and informed consent

The study has been approved by Peking University First Hospital (2018-R-141, PUFH) and University of Birmingham (ERN_18-1177, UoB). Residents responding to the study invitation will be given the study information sheet with enough time to read it and will have opportunity to ask the researcher any questions about the study. Interested respondents who are eligible for the study will be asked to sign a consent form, or if unable to consent, a family member will be asked to sign on their behalf. Consent will also be sought to allow the research team to contact participants about future studies related to the Breathe Well program; this is optional and will not affect eligibility for the study described in this paper.

Dissemination and Publication policy

For peer review only

Study results will be published in peer-reviewed journals and presented at national and international conferences, as well as relevant community/stakeholder engagement activities. Participants who explicitly express a wish to be informed about the research outcome will be contacted and offered to receive an article or poster with a lay summary of the study.

Regulatory issues

Funding

The NIHR Global Health Research (GHR) Programme is funded through UK Official Development Assistance (ODA) via the Department of Health and Social Care (DHSC). This research was commissioned by the National Institute for Health Research (NIHR) GHR Programme using UK aid from the UK government.

Indemnity

The study is not an intervention study, and as such poses low risk to participants. However, clinical insurance was purchased in case of Serious Adverse Events (SAEs).

Patient and public involvement

The research team conducted a research prioritization exercise with patients, clinicians and policy makers, and the need to identify effective screening strategies for undiagnosed COPD was one of the research areas prioritized. All stakeholders involved in this exercise will receive study updates twice a year, will be kept informed of findings and will be consulted at the end of the study regarding implications for practice and policy decisions, as well as advice on appropriate dissemination of study findings.

A patient advisory group (PAG) has been set up, which is funded to meet at approximately quarterly intervals or according to need, and will advise on a range of aspects of the design, conduct, analysis and dissemination of the study. The PAG will discuss issues as requested by the CIs and the chair will report their comments back to the investigators.

In addition, the study has a Trial Steering Committee (TSC) that meets regularly and comprises various independent members, including a patient and a clinician representative as well as international experts in respiratory research. The TSC also includes several members of the study research team.

Sponsor

Prof Chunhua Chi, who is the Director, Department of General Practice, Peking University First Hospital, will act as the main sponsor for this study.

Discussion

This study aims to identify the most effective and cost-effective screening strategies for identifying undiagnosed COPD in the primary care setting in China.

To the best of our knowledge, this is the first study to assess the accuracy of different COPD screening strategies including screening questionnaires, peak flow and microspirometer measurement. This study is being conducted in a range of community hospitals from rural and urban areas which are broadly representative of primary care institutions in China. The planned cost-effectiveness analysis will calculate the cost per true case detected for each strategy, which

For peer review only

will help inform decisions about the future feasibility of screening strategies within the primary care setting in China. This trial should inform primary care across China and elsewhere with similar healthcare systems, and help to direct current effort towards case-finding more efficiently.

This study also helps building research capacity within primary care, as it is the first respiratory study for the participating community hospitals and the majority of General Practioner(GP) researchers being taught how to conduct high quality spirometry will have no prior experience and might have difficulty in understanding the research process.

Recent health policies have seen lung function testing being incorporated into a routine health examination programme amongst the general population , and objectives being set to increase the proportion of those over 40 years old received lung function tests from 7.1% in 2017 to 15% in 2020 and 25% in 2025[38]. Considering the increasing importance of lung function testing in China and the intensive spirometry training given to clinicians through this study, we believe this study could also help improve the quality of COPD management in primary care in China.

Considering that there is no “GP first contact” in China yet, it is challenging to plan how best to attract people attending community hospitals and recruit them into the study. However, voluntary pulmonary function screening identifies high rates of undiagnosed asymptomatic COPD[7]. How to encourage residents to volunteer to participate in screening is also something we need to consider. It is also hard to anticipate residents’ willingness to participate in this study and how participants will respond to the study measures. However, what is worth mentioning, besides posters, referred by doctors, friends or family members, Wechat, a social media which has a prominence in Chinese society now, also plays an important role in the recruitment process to inform residents or disseminate the programme. Last but not least, it will be important to discuss how this approach can be rolled out from a trial setting into routine practice. Real world study may be the most appropriate method to make it clear how the validated screening strategy works in practices.

Conclusion

COPD screening is extremely important to China and its 99.9 million potential COPD patients[5]. This trial will provide robust evidence about the effectiveness and cost-effectiveness of different COPD screening methods and strategies and confirm which the best COPD screening strategy is. The service might be a template for delivery of a procedural screening strategy that can reach large numbers of an under recognized population. Although the long-term benefits of screening are still to be proven, this programme has capacity to contribute significantly to improving public health.

Additional file

Additional file: Appendix 1. Study questionnaire. **Appendix 2**. Screening questionnaire. **Appendix 3**. Health economic questionnaire. **Appendix 4**. Patient informed consent. **Appendix 5.** Ethic approvals

Abbreviations

COPD: Chronic obstructive pulmonary disease

CHSC: Community Health Service Center

GP: General Practitioner

For peer review only

CRF: case report form

PUFH: Peking University First Hospital

UoB: University of Birmingham

NIHR: National Institute for Health Research

UK: the United Kingdom.

PAG: Patient Advisory Group

TSC: Trial Steering Committee

GHR: The NIHR Global Health Research

ODA: Official Development Assistance funding

DHSC: the Department of Health and Social Care

Competing interests

The authors declare that they have no competing interests.

Authors' contributions

Zihan Pan and Andrew P Dickens wrote the protocol paper with input from all other authors. Rachel E Jordan led the design of the trial, with contributions and advice from all other investigators. Chunhua Chi, Xia Kong, Peymane Adab, KK Cheng contributed to decisions on outcome measures. Chunhua Chi and KK Cheng advised on involving GP practices, Rachel E Jordan, Peymane Adab, Alexandra Enocson and Andrew P Dickens advised on lung function testing. Andrew P Dickens and Rachel E Jordan designed the intervention. Alice Sitch and Sue Jowett designed the analysis plan and economic evaluation. Chunhua Chi was the local PI. All authors have read and approved the final draft.

Acknowledgements

The views expressed in this publication are those of the author(s) and not necessarily those of the NIHR or the Department of Health and Social Care. We gratefully acknowledge International Primary Care Respiratory Group (IPCRG) for introducing us to the primary care networks involved in this study and for its continued facilitation of clinical engagement. This paper presents independent research supported by the NIHR Birmingham Biomedical Research Centre at the University Hospitals Birmingham NHS Foundation Trust and the University of Birmingham.

Author details

1. Department of General Practice, Peking University First Hospital, Beijing, China
2. Institute of Applied Health Research, University of Birmingham, Birmingham, UK.
3. NIHR Birmingham Biomedical Research Centre, University Hospitals Birmingham NHS Foundation Trust and University of Birmingham, UK

For peer review only

References

[1] Global Initiative for Chronic Obstructive Lung Disease (GOLD).Global strategy for the diagnosis, management, and prevention of chronic obstructive pulmonary disease (2018 report).https://goldcopd.org/wp-content/uploads/2018/11/GOLD-2019-v1.7-FINAL-14Nov2018-WMS.pdf.

[2] Lozano R, Naghavi M, Foreman K, et al. Global and regional mortality from 235 causes of death for 20 age groups in 1990 and 2010: a systematic analysis for the Global Burden of Disease Study 2010. Lancet. 2012 Dec 15;380(9859):2095-128.

[3] López-Campos JL, Tan W, Soriano JB. Global burden of COPD. Respirology. 2016. 21(1): 14-23.

[4] Bednarek M, Maciejewski J, Wozniak M, Kuca P, Zielinski J. Prevalence, severity and underdiagnosis of COPD in the primary care setting. Thorax. 2008. 63(5): 402-7.

[5] Wang C, Xu J, Yang L et al. Prevalence and risk factors of chronic obstructive pulmonary disease in China (the China Pulmonary Health [CPH] study): a national cross-sectional study. Lancet. 2018 Apr 28;391(10131):1706-1717.

[6] Hangaard S, Kronborg T, Hejlesen OK. Characteristics of Subjects With Undiagnosed COPD Based on Post-Bronchodilator Spirometry Data. Respir Care. 2019. 64(1): 63-70.

[7] Wang S, Gong W, Tian Y. Voluntary pulmonary function screening identifies high rates of undiagnosed asymptomatic chronic obstructive pulmonary disease. Chron Respir Dis. 2016. 13(2): 137-43.

[8] Lu M, Yao WZ, Zhong NS,et al. Asymptomatic patients of chronic obstructive pulmonary disease in China. Chin Med J (Engl). 2010 Jun;123(12):1494-9.

[9] US Preventive Services Task Force (USPSTF). Screening for Chronic Obstructive Pulmonary Disease: US Preventive Services Task Force recommendation statement. JAMA 2016; 315: 1372 – 77.

[10] Bao HL, Cong S, Wang N, et al. Survey and analyses of population at high risk of chronic obstructive pulmonary disease in China,2014. Chin J Epidemiol, May 2018, Vol.39, No.5.(in Chinese)

[11] Fang L, Gao P, Bao H, et al. Chronic obstructive pulmonary disease in China: a nationwide prevalence study. Lancet Respir Med. 2018. 6(6): 421-430.

[12] National Health and Family Planning Commission of the People's Republic of China.The 13th Five-Year Plan for Healthcare. Dec 27, 2016. http://www.gov.cn/zhengce/content/2017-01/10/content_5158488.htm. (in Chinese).

[13] Wen FQ, Shen YC, Chen .Pulmonary function test in the management of chronic obstructive pulmonary disease patients in China: challenges and countermeasures. Chin J Tuberc Respir Dis, December 2017, Vol.40, No.12.(in Chinese)

[14] He QY. Standardized lung function measurement technology to improve the lung function determination. ChinJTubercRespirDis, December2006, Vol.29, No.12.(in Chinese)

[15] Calverley PM, Nordyke RJ, Halbert RJ, Isonaka S, Nonikov D. Development of a population-based screening questionnaire for COPD. COPD. 2005. 2(2): 225-32.

[16] Price DB, Tinkelman DG, Halbert RJ, et al. Symptom-based questionnaire for identifying COPD in smokers. Respiration. 2006. 73(3): 285-95.

[17] Martinez FJ, Mannino D, Leidy NK, et al. A New Approach for Identifying Patients with Undiagnosed Chronic Obstructive Pulmonary Disease. Am J Respir Crit Care Med. 2017 Mar 15;195(6):748-756.

[18] Zhou YM, Chen SY, Tian J,et al. Development and validation of a chronic obstructive pulmonary disease screening questionnaire in China. Int J Tuberc Lung Dis. 2013 Dec;17(12):1645-51.

[19] Martinez FJ, Raczek AE, Seifer FD, et al. Development and initial validation of a self-scored COPD Population Screener Questionnaire (COPD-PS). COPD. 2008. 5(2): 85-95.

[20] Represas-Represas C, Fernández-Villar A, Ruano-Raviña A, Priegue-Carrera A, Botana-Rial M. Screening for Chronic Obstructive Pulmonary Disease: Validity and Reliability of a Portable Device in Non-Specialized Healthcare Settings. PLoS One. 2016. 11(1): e0145571.

[21] Kjeldgaard P, Lykkegaard J, Spillemose H, Ulrik CS. Multicenter study of the COPD-6 screening device: feasible for early detection of chronic obstructive pulmonary disease in primary care. Int J Chron Obstruct Pulmon Dis. 2017. 12: 2323-2331.

[22] Van den Bemt L, Wouters BC, Grootens J,et al. Diagnostic accuracy of pre-bronchodilator FEV1/FEV6 from microspirometry to detect airflow obstruction in primary care: a randomised cross-sectional study. NPJ Prim Care Respir Med. 2014 Aug 14;24:14033.

[23] Kim JK, Lee CM, Park JY, et al. Active case finding strategy for chronic obstructive pulmonary disease with handheld spirometry. Medicine (Baltimore). 2016. 95(50): e5683.

[24] Labor M, Vrbica Ž, Gudelj I, Labor S, Plavec D. Diagnostic accuracy of a pocket screening spirometer in diagnosing chronic obstructive pulmonary disease in general practice: a cross sectional validation study using tertiary care as a reference. BMC Fam Pract. 2016. 17(1): 112.

[25] Jackson H, Hubbard R. Detecting chronic obstructive pulmonary disease using peak flow rate: cross sectional survey. BMJ. 2003. 327(7416): 653-4.

[26] Tian J, Zhou Y, Cui J, et al. Peak expiratory flow as a screening tool to detect airflow obstruction in a primary health care setting. Int J Tuberc Lung Dis. 2012. 16(5): 674-80.

[27] Sogbetun F, Eschenbacher WL, Welge JA, Panos RJ. A comparison of five surveys that identify individuals at risk for airflow obstruction and chronic obstructive pulmonary disease. Respir Med. 2016. 120: 1-9.

[28] Van Schayck CP, Halbert RJ, Nordyke RJ, Isonaka S, Maroni J, Nonikov D. Comparison of existing symptom-based questionnaires for identifying COPD in the general practice setting. Respirology. 2005. 10(3): 323-33.

[29] Spyratos D, Haidich AB, Chloros D, Michalopoulou D, Sichletidis L. Comparison of Three Screening Questionnaires for Chronic Obstructive Pulmonary Disease in the Primary Care. Respiration. 2017. 93(2): 83-89.

[30] Tsukuya G, Samukawa T, Matsumoto K, et al. Comparison of the COPD Population Screener and International Primary Care Airway Group questionnaires in a general Japanese population: the Hisayama study. Int J Chron Obstruct Pulmon Dis. 2016. 11: 1903-9.

[31] Bossuyt, P. M. et al. STARD 2015: an updated list of essential items for reporting diagnostic accuracy studies. BMJ 351, h5527, doi:10.1136/bmj.h5527 (2015).

[32] Zhong N, Wang C, Yao W, et al. Prevalence of chronic obstructive pulmonary disease in China: a large, population-based survey. Am J Respir Crit Care Med 2007; 176: 753 – 60.

[33] Frith P, Crockett A, Beilby J, et al. Simplified COPD screening: validation of the PiKo-6® in

For peer review only

primary care. Prim Care Respir J. 2011. 20(2): 190-8, 2 p following 198.

[34] Stanley AJ, Hasan I, Crockett AJ, van Schayck OC, Zwar NA. COPD Diagnostic Questionnaire (CDQ) for selecting at-risk patients for spirometry: a cross-sectional study in Australian general practice. NPJ Prim Care Respir Med. 2014. 24: 14024.

[35] Zhang Q, Wang M, Li X, Wang H, Wang J. Do symptom-based questions help screen COPD among Chinese populations. Sci Rep. 2016. 6: 30419.

[36] A Guide to Performing Quality Assured Diagnostic Spirometry. http://www.artp.org.uk/en/professional/artp-tandards/index.cfm/QADS%20Apr%202013.

[37] Alonzo TA, Pepe MS, Moskowitz CS. Sample size calculations for comparative studies of medical tests for detecting presence of disease. Stat Med. 2002. 21(6): 835-52.

[38] Medium-long term plan of prevention and treatment for chronic diseases (2017-2025, in Chinese) http://www.gov.cn/xinwen/2017-02/14/content_5167942.htm.(in Chinese)

For peer review only

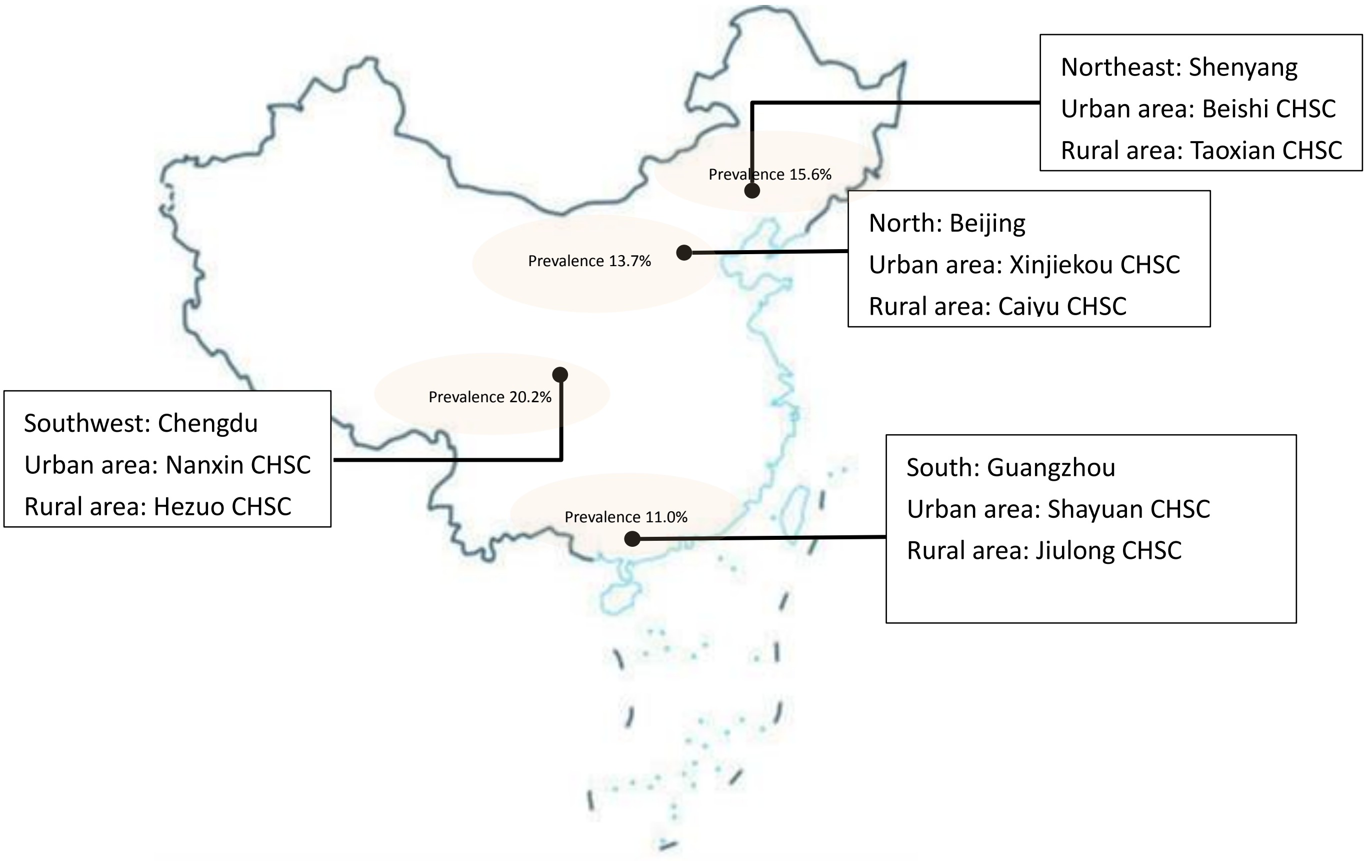

Figure 1 the map of Breathe Well-China research sites

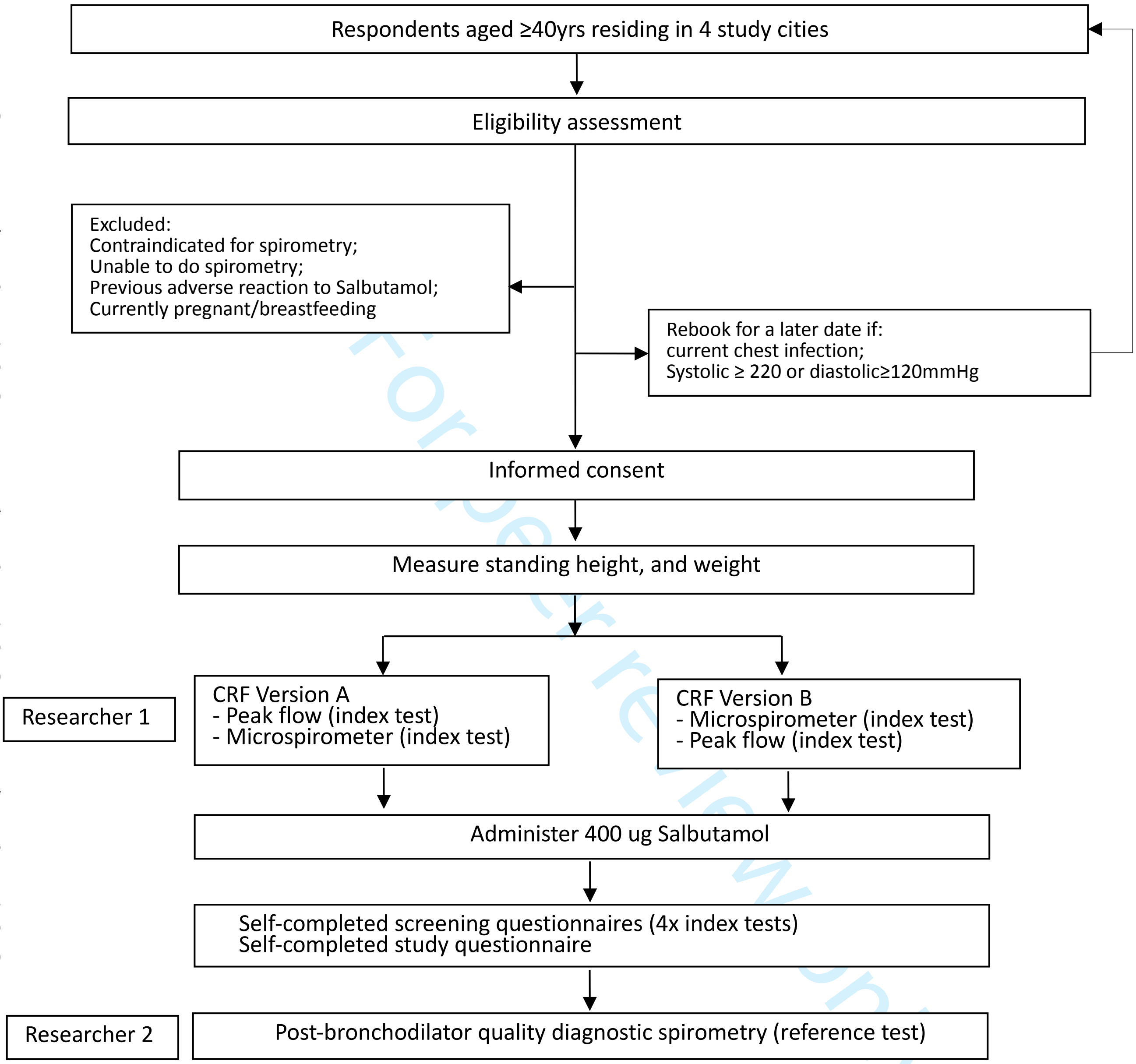

Figure 2 flow of participants

For peer review only - http://bmjopen.bmj.com/site/about/guidelines.xhtml

中国慢阻肺筛查策略评估：健康呼吸 Breathe Well 研究项目

Evaluating screening strategies for identifying undiagnosed COPD in China: a Breathe Well project

调查问卷

Study Questionnaire

For peer review only

研究对象编号<br>Patient Initials	
问卷编号<br>Study ID	
填写日期<br>Date	
研究人员编号<br>Interviewer ID	

您的回答和意见对们很有价值。请您在翻页之前阅读以下内容，非常感谢您的合作！

Your answers and opinions are valuable to us. We would be very grateful if you could read the below before turning the page:

- **如有可能，请您自行填写这份问卷。**

  Please complete this questionnaire yourself if at all possible
- **请尽可能回答所有问题**

  Please answer all questions as well as you can
- **请不要花太多时间思考您的回答**

  Do not spend too long thinking about your answers
- **如果有人替您回答了这份问卷，他们需要记录下您的答案**

  If someone is completing this on your behalf, they should record your answers

调查问卷 v7 2018.10.4
Study questionnaire 041018

1. 性别
Sex

☐ 男
Male

☐ 女
Female

2．年龄
What is your age?

岁
__________ years old

3．您获得的最高学历是什么？
What is the highest level of qualification that you have?

没有正式的学历 ☐
No formal qualification

低于高中水平 ☐
Less than High school

高中水平 ☐
High school

大专
Junior college

本科 ☐
Bachelor

研究生 ☐
Master

博士 ☐
Doctor

4. 您的工作状态是什么样的？
What is your employment status?

调查问卷 v7 2018.10.4
Study questionnaire 041018

For peer review only

个体
Self-employed ☐

受雇于工作单位
Employed ☐

无工作
Unemployed ☐

退休
Retired ☐

5. 您绝大部分时间生活在哪里？
Where have you spent most of your life?

城市
Urban areas ☐

农村
Rural areas ☐

6. 您目前的吸烟状态是？
What is your current smoking status?

当前吸烟者（每天至少吸 1 支，至少吸了 6 个月） ☐
Current smoker (smoke at least 1 cigarette per day for at least the last 6 months)

既往吸烟者（既往每天至少吸 1 支，至少吸了 6 个月，但是现在不吸了） ☐
Ex-smoker (previously smoked at least 1 cigarette per day for at least 6 months, but not now)

我从不经常性地吸烟 （**如果您选择了这个选项，请跳至第 9 题**） ☐
I have never smoked regularly （***please go to question 10***）

7. 如果您曾经吸过烟，那么您是几岁**开始**经常性地吸烟？（“经常性地吸烟”指的是，至少 1 支/每天或者 7 支/每周，至少 6 个月）
If you have ever smoked, at about what age did you **start** to smoke regularly? (by regularly we mean at least 1 cigarette/day or 7 cigarettes/week for at least 6 months)
________ 岁

For peer review only - http://bmjopen.bmj.com/site/about/guidelines.xhtml

_________ years old

如果您曾经吸过烟，您是从什么时候**停止**经常性地吸烟的？
If you are an ex-smoker, at what age did you **stop** smoking regularly?
_________ 岁
_________ years old

8. 目前您每天常常吸多少支烟？或者，当您是烟民的时候，您是否经常性地吸烟？
How much do you usually smoke each day now, or did you usually smoke when you were a smoker?

电子烟<br>Electronic cigarettes (or e-cigarettes)		支/天<br>number/day
过滤嘴型香烟<br>Filter cigarettes		支/天<br>number/day
无过滤嘴/手卷烟<br>Non-filter/hand rolled cigarettes		支/天<br>number/day
雪茄<br>Cigars		支/天<br>number/day
烟斗<br>Pipe tobacco		烟草…克/天<br>g/day tobacco

9. 您的整体健康状况如何?
How is your health in general?
非常好 Very Good☐ 好 Good☐ 一般 Fair☐ 差 Bad☐ 非常差 Very Bad☐

10. 患病情况
Medical conditions

您患有以下疾病吗？请选择
Has a doctor EVER told you that you had any of the following conditions? Please tick all that apply

疾病<br>Conditions	有<br>Yes	无<br>No
慢性阻塞性肺疾病<br>Chronic Obstructive Pulmonary Disease		
慢性支气管炎/肺气肿<br>Chronic bronchitis/emphysema		

调查问卷 v7 2018.10.4
Study questionnaire 041018

For peer review only

哮喘<br>Asthma		
结核<br>Tuberculosis		
高血压<br>Hypertension		
糖尿病<br>Diabetes Mellitus		
胃食管返流<br>GERD		
焦虑<br>Anxiety		
抑郁<br>Depression		
心脏病<br>Heart disease		
癌症<br>Cancer		

11. 当您在水平地面上行走或在一个小山坡上行走时，您是否因呼吸急促而感到困扰？

Are you troubled by shortness of breath when hurrying on the level ground or walking up a slight hill?

是 Yes ☐ 否 No ☐

12. 您在平地上和同龄人一起行走时，您是否会感到气促？

Do you get short of breath walking with other people of your own age on level ground?

是 Yes ☐ 否 No ☐

13. 当您在平地上按自己的速度行走时，您是否会因为呼吸而不得不停下来？

Do you have to stop for breath when walking at your own pace on level ground?

是 Yes ☐ 否 No ☐

14. 当您在平地上行走 100 米或几分钟后，您是否会因为呼吸而不得不停下来？

Do you have to stop for breath after walking for 100yds (or after a few minutes) on the level?

是 Yes ☐ 否 No ☐

15. 您是否因呼吸困难而不能离开家或者您是否在穿衣服或脱衣服的时候有呼吸困难？

Are you too breathless to leave the house or are you breathless when dressing or undressing?

是 Yes ☐ 否 No ☐

For peer review only - http://bmjopen.bmj.com/site/about/guidelines.xhtml

For peer review only

COPD 评估 (CAT)

COPD Assessment Test (CAT)

即使您没有肺部问题，也请完成以下问卷。

Please complete the below questionnaire even if you do not have a lung condition

16. 您肺部的问题怎么样？对于下面的每个项目，请在 0-5 中圈出最符合您的情况的分数。

How are your lung problems? For each item below place a mark in the box that best describes your experience on a scale of 0-5

例如：我极开心 | 0 | ✓ 1 | 2 | 3 | 4 | 5 | 我极不开心

Example: I am very happy | 0 | √ 1 | 2 | 3 | 4 | 5 | I am very sad

我从不咳嗽<br>I never cough	0	1	2	3	4	5	我总是咳嗽<br>I cough all the time
我肺里一点痰也没有<br>I have no phlegm (mucus) in my chest at all	0	1	2	3	4	5	我肺里有很多很多痰<br>My chest is completely full of phlegm (mucus)
我一点也没有胸闷的感觉<br>My chest does not feel tight at all	0	1	2	3	4	5	我有很重的胸闷的感觉<br>My chest feels very tight
当我在爬坡或爬一层楼时，我并不感觉喘不过气来<br>When I walk up a hill or one flight of stairs I am not breathless	0	1	2	3	4	5	当我爬坡或爬一层楼时，我感觉非常喘不过气来<br>When I walk up a hill or one flight of stairs I am very breathless
我在家里的任何劳动都不受慢阻肺的影响<br>I am not limited doing any activities at home	0	1	2	3	4	5	我在家里的任何劳动都很受慢阻肺的影响<br>I am very limited doing activities at home
尽管我有肺病，我还是有信心外出<br>I am confident leaving my home despite my lung condition	0	1	2	3	4	5	因为我有肺病，对于外出我完全没有信心<br>I am not at all confident leaving my home because of my lung condition

调查问卷 v7 2018.10.4

Study questionnaire 041018

For peer review only

我睡得好<br>I sleep soundly	0 | 1 | 2 | 3 | 4 | 5	因为我有肺病，我睡得不好<br>I don't sleep soundly because of my lung condition
我精力旺盛<br>I have lots of energy	0 | 1 | 2 | 3 | 4 | 5	我一点精力都没有<br>I have no energy at all

COPD 评估测试和 CAT 的标志是 GlaxoSmithKline 集团公司的商标。
©2009 GlaxoSmithKline 集团公司。版权所有。

17. 您在儿童时期，患过气管炎，肺炎或者严重的百日咳吗？

Did you ever have bronchitis, pneumonia or severe whooping cough as a child?

☐ 有
Yes

☐ 无 如果没有，跳至第 19 题
No If no, ***please go to question 19***

18. 如果有，您患病的时候多大？（或者首次发作的时候）？

If yes, approximately how old were you when you had this (or first time if several episodes)?

___岁
_____years
或
or
______月
_____months

19. 您在孩童时期，是否患过肺结核？

Did you ever have tuberculosis as a child?

☐ 是
Yes

☐ 否，如果否，请跳至第 21 题
No If no, ***please go to question 21***

20. 如果是，那时您大概多大？（或者第一次患病多大，如果有复发的话）

If yes, approximately how old were you when you had this (or first time if several episodes)?

_____年
_____years
或
Or
______月
______months

For peer review only - http://bmjopen.bmj.com/site/about/guidelines.xhtml

21. 以下哪些化学物质或颗粒是您目前正在工作/家中接触的，或者您在工作/家中已经接触过了哪些？(生物质燃料包括木柴，粪肥，农作物残留物如秸秆/草/灌木，煤和煤油)

Which of the following chemicals or particulates are you currently exposed to at work/home, or which have you been exposed to at work/home in the past? (Biomass fuel consists of fire wood, manure, agricultural crop residues such as straw/grass/shrubs, coal fuels and kerosene)

物质种类<br>chemicals or particulates	是，正在接触<br>Yes, currently	是，过去接触过<br>Yes, in the past	否，从没接触过<br>No, never
烹饪油烟<br>Cooking fumes			
生物质燃料<br>Biomass fuel			
各种物质的蒸汽<br>Steam of various substances			
气体<br>Gas			
灰尘<br>Dust			

22. 如果您接触过上述物质，您接触了多少年？

If you ticked 'yes' to any exposures, how many years have you been exposed to them?

________年

______years

23. 如果您接触了烹饪油烟或者生物质燃料，您的家中/工作地点有烟囱或排烟系统吗？

If exposed to cooking fumes or biomass fuels, did the home/workplace have a chimney or exhaust system?

☐ 有

Yes

☐ 无

No

非常感谢您抽出宝贵的时间参与本研究！

Thank you for taking the time to complete this survey

调查问卷 v7 2018.10.4

Study questionnaire 041018

For peer review only

Evaluating screening strategies for identifying undiagnosed COPD in China: a Breathe Well project

中国慢阻肺筛查策略评估：健康呼吸 Breathe Well 研究项目

For peer review only

Lung health questionnaire

肺部健康问卷

Participant Initials<br>研究对象编号	
Study ID<br>问卷编号	
Date<br>填写日期	
Interviewer ID<br>研究人员编号	

For peer review only - http://bmjopen.bmj.com/site/about/guidelines.xhtml

Some questions in the following booklets may appear similar. However, it is important that we ask these questions in slightly different ways so please complete all questions, answering them as accurately as possible.

一些问题可能相似，但是我们以稍微不同的方式提出这些问题很重要。

因此，请您完成所有的问题，并尽可能准确地作答。

CDQ

1. Age group, years
   年龄

40–49 ☐ 50-59 ☐ 60-69 ☐ 70+ ☐

2. What is your weight in kilograms?
   您的体重（公斤）？

   _____________kilograms
   _____________ 公斤

   What is your height in meters?
   您的身高（米）？

   _____________ metres
   _____________ 米

3. Smoking
   吸烟强度，包年
   What is the total number of years you have smoked?
   您一共吸烟多少年？
   _____ years

   _____ 年

   How many cigarettes do you currently smoke each day (or 'did smoke each day' if ex-smoker)?
   目前您每天吸多少支烟？（或，如果是既往吸烟者，过去您每天吸多少支烟？）
   _____ cigarettes

   _____ 支

4. Does the weather affect your cough?
   您的咳嗽是否受天气影响？

Yes ☐ No ☐

For peer review only - http://bmjopen.bmj.com/site/about/guidelines.xhtml

For peer review only

是 ☐ 否 ☐

5. Do you ever cough up phlegm (sputum) from your chest when you don't have a cold?
   您不感冒的时候，会从胸腔里咳出痰吗？（区别于从嗓子中咳痰）

Yes ☐ No ☐
是 ☐ 否 ☐

6. Do you usually cough up phlegm (sputum) from your chest first thing in the morning?
   清晨您的第一件事是从胸腔里咳出痰吗？

Yes ☐ No ☐
是 ☐ 否 ☐

7. How frequently do you wheeze?
   您喘息的次数是多少？

Occasionally or more often ☐ Never ☐
有时候或更频繁 ☐ 从不 ☐

8. Do you have or have you had any allergies?
   目前或既往您有过敏物吗？

Yes ☐ No ☐
是 ☐ 否 ☐

CAPTURE

1. Have you ever lived or worked in a place with dirty or polluted water or air, smoke or second-hand smoke or dust?
   您是否曾经在有脏的或受到污染的水或空气，烟雾或二手烟雾或灰尘的地方生活或工作？

Yes ☐ No ☐
是 ☐ 否 ☐

2. Does your breathing change with seasons, weather or air quality?
   您的呼吸是否随着季节、天气或空气质量而变化？

Yes ☐ No ☐
是 ☐ 否 ☐

3. Does your breathing make it difficult to do things such as carry heavy loads, shovel dirt or snow, jog, play tennis or swim?
   您的呼吸是否会使您难以进行一些工作，比如提重物，铲土或积雪，慢跑，打网球或游泳等？

For peer review only - http://bmjopen.bmj.com/site/about/guidelines.xhtml

For peer review only

Yes ☐ No ☐
是 ☐ 否 ☐

4. Compared to others your age, do you tire easily?
和您的同龄人相比，您是否容易感到疲劳？

Yes ☐ No ☐
是 ☐ 否 ☐

5. In the past 12 months, how many times did you miss work, school, or other activities due to a cold, bronchitis, or pneumonia?
在过去的 12 个月里，您有多少次因感冒、支气管炎或肺炎而错过了工作、学校或其他活动？

0 ☐ 1 ☐ 2 or more ☐
0 ☐ 1 ☐ 2 或以上 ☐

Copyright© 2015 by Cornell University, University of Kentucky, and Evidera. All Rights Reserved
版权所有©2015 康奈尔大学，肯塔基大学和 Evidera。版权所有

Symptom-based questionnaire

1. How frequently are you exposed to second-hand smoking?
您接触二手烟的频率是多少？

<7hrs per week ☐ ≥7hrs per week ☐

＜7小时/周 ☐ ＞7 小时/周 ☐

2. Do you often cough when you do not have a cold?
您是否在不感冒的时候经常咳嗽？

Yes ☐ No ☐
是 ☐ 否 ☐

3. Do you have more signs of shortness of breath compared with others of the same age?
和同龄人相比，您是否有更多的呼吸急促的症状？

Yes ☐ No ☐
是 ☐ 否 ☐

4. Have you had long-term exposure to dust or chemical particles?
您是否长期地接触粉尘或化学颗粒？

Yes ☐ No ☐

For peer review only - http://bmjopen.bmj.com/site/about/guidelines.xhtml

是 ☐ 否 ☐

5. Did you have a history of chronic respiratory diseases when you were a child?
   在您孩童时期，您是否有慢性呼吸疾病的病史？

Yes ☐是 No ☐
是 ☐ 否 ☐

COPD-SQ

1. Do you often cough?
   您是否经常咳嗽？

Yes ☐ No ☐
是 ☐ 否 ☐

2. Family history of respiratory disease
   是否有呼吸疾病家族史？

Yes ☐ No ☐
是 ☐ 否 ☐

3. Exposure to biomass smoke from cooking fires
   是否接触烹饪产生的生物烟雾？

Yes ☐ No ☐
是 ☐ 否 ☐

For peer review only

Study ID [ ][ ][ ][ ][ ][ ]

COPD case finding study: assessment of task timing

IMPORTANT: Please write how long each task takes in minutes.

Assessment station 1 – NO TIMING REQUIRED

Assessment station 2

Please only note the time for standing height (not arm span or weight)

Standing height **start time** ______ **end time** ______ [ ][ ] minutes

Assessment station 3

Pre-bronchodilator peak flow **start time** ______ **end time** ______ [ ][ ] minutes

Pre-bronchodilator microspirometry **start time** ______ **end time** ______ [ ][ ] minutes

Assessment station 4

Administration of Salbutamol **start time** ______ **end time** ______ [ ][ ] minutes

Assessment station 5

Completion of Lung Health questionnaire (CDQ etc) **start time** ______ **end time** ______
[ ][ ] minutes

Did the patient require assistance? Yes [ ] No [ ]

If yes, was assistance required for the whole questionnaire? Yes [ ] No [ ]

Assessment station 6

Post-bronchodilator spirometry **start time** ______ **end time** ______ [ ][ ] minutes

For peer review only

版本号：6.0 版本日期：2018.10.25

中国慢阻肺筛查策略评估：健康呼吸 Breathe Well 研究

知情同意书

我们正在开展"慢性阻塞性肺疾病不同筛查策略在中国四个城市≥40 岁人群中的有效性及成本-效益的评价性研究"研究，因您的情况符合入组条件，我们邀请您参加本项研究。本知情同意书将向您介绍本项研究的目的、步骤、获益和风险等，请仔细阅读后决定是否参加。当研究者向您说明和讨论知情同意书时，您可以随时提问并要求他/她向您解释不明白的地方。您可以与家人、朋友以及您的经治大夫讨论之后再做决定。

本项研究由英国伯明翰大学发起，在中国的项目负责人是北京大学第一医院迟春花主任医师。

1. 为什么进行本项研究？

慢性阻塞性肺疾病（简称"慢阻肺"）为国家带来了巨大的经济负担，也为患者带来了心理和经济的重创。中国具有庞大的慢阻肺患者群，然而却面临着漏诊率高、诊断不及时的问题。社区是慢性非传染性疾病防治的重要关卡，而社区在早期发现慢阻肺人群、及早对慢阻肺人群进行干预方面有着独特的优势。然而目前，什么样的筛查方式是有效的并且能够早期发现慢阻肺患者的方式尚不明确。鉴于此，我们设计了此研究，以评估在中国的社区中不同慢阻肺筛查方式在≥40 岁人群中的筛查准确性。通常用于测量肺功能的仪器叫做"肺量计"，我们会将其与筛查测试，即微型肺量计，呼气峰流速及筛查问卷进行对比。

2. 哪些人将被邀请参加本项研究？

入选标准：

1）≥40 岁

For peer review only - http://bmjopen.bmj.com/site/about/guidelines.xhtml

2）出于任何原因去社区卫生服务中心/卫生院的人群

3）居住在本社区卫生服务中心/卫生院附近

排除标准：

1）不能配合进行肺功能检查（如，痴呆，牙齿缺损而不能形成良好的闭合等）

2）有肺功能检查禁忌症：过去 3 周有胸部感染，过去 1 个月有咯血，严重的心绞痛，未能控制的高血压，气胸，或过去 3 个月有肺结核、心脏病发作、视网膜脱离或胸部/腹部/脑/耳朵/眼部手术病史

3）怀孕、哺乳期妇女

4）既往对沙丁胺醇有不良反应

此外，研究人员将根据您的实际情况来判断您是否适合参加本项研究。

3．多少人将参与本项研究？

本项研究计划招募约 2000 名受试者，本中心需招募约 250 名受试者。

4. 本项研究包括哪些内容？

本研究为多中心的横断面调查（诊断试验准确性研究）。研究地点为北京（北方）、成都（西南）、广州（南方）、沈阳（东北）。每个城市各选取 2 个研究地点：1 个城区的社区卫生服务中心，1 个郊区的社区卫生服务中心/卫生院。

仪器型号及药品信息：

仪器：

For peer review only - http://bmjopen.bmj.com/site/about/guidelines.xhtml

1）呼气峰流速仪 PEF：尖峰吐气流量计（USPE，崇仁（厦门））

2）微型肺量计：COPD-6

3）肺量计：The ndd Easy On-PC （ndd Medizintechnik AG）

药品：

支气管扩张剂：沙丁胺醇气雾剂（葛兰素史克公司）

本研究中所使用的仪器及药品均已获得国家食品药品监督管理总局（CFDA）批准。

研究评估：

如果您参加该研究，我们将对您进行一次研究评估，约 90 分钟。如果您符合我们的研究标准，我们将测量您的身高和体重，然后按要求进行三次吹气测试，并完成研究问卷。

本研究的数据采集流程包括以下内容：

1）选择研究对象，签署知情同意：研究人员将根据入选标准和排除标准确定您是否能够参与我们的研究，并检查您目前是否有胸部感染（如果有急性的胸部感染，将重新预约）。如果您符合我们的研究入选标准，您可以向研究人员询问任何有关本研究的事项，然后签署知情同意书。除了同意主要研究外，您还会被询问是否同意研究人员联系您以便参与与该研究相关的未来的研究。参与未来的研究是可选的，不会影响您参与本次研究。

2）测量身高，体重；

3）测量呼气峰流速（FEFR），进行微型肺量计检查：您将使用两种仪器进行吹气测

For peer review only - http://bmjopen.bmj.com/site/about/guidelines.xhtml

试，一种仪器叫呼气峰流速仪，另一种仪器叫微型肺量计。您将使用每个仪器进行 3 次吹气，详情如下所述。该两项检查不使用沙丁胺醇。

肺功能（呼气峰流速）：研究人员将使用呼气峰流速仪（尖峰吐气流量计（USPE，崇仁（厦门）））记录(呼气峰流速)PEF;

肺功能（微型肺量计）：使用微型肺量计（如 COPD-6）测量 FEV_1(一秒用力呼气容积)，FEV_6（六秒用力呼气容积），系统自动计算得出 FEV_1/FEV_6 比值。男性 FEFR＜3501/min，女性 FEFR＜2501/min 为异常。FEV_1/FEV_6 ＜0.75，FEV_1/FEV_6 ＜0.78 结果为阳性。研究人员记录 FEV_1 和 FEV_6 值最高值，以及此时的 FEV_1/FEV_6 比值。

4）使用支气管扩张剂：您将吸入每喷 100μg 共 4 喷（400μg）沙丁胺醇。具体流程如下：将沙丁胺醇的喷嘴与储雾罐接口相连，然后将一次性咬嘴与储雾罐另一端接口相连，用嘴将咬嘴另一端包严，然后将沙丁胺醇（1 喷为 100ug）共 4 喷（400μg），喷入储雾罐中，此时用嘴呼吸，通过咬嘴进行 3 次深吸气、深呼气。20 分钟后进行吸入支气管扩张剂后肺功能的测定。

5）答问卷：您将独自完成四份慢阻肺筛查问卷，分别是，CAPTURE，CDQ，基于症状的筛查问卷以及来自 COPD-SQ 的部分问题。

此外，您还将完成一份调查问卷，调查问卷内容包括：人口学统计数据（性别，年龄，婚姻状况，受教育程度，生活方式（吸烟状况，锻炼），暴露（生物质烟雾，化学品和微粒的职业暴露），健康（医学诊断[包括慢阻肺，哮喘，结核等]，合并症，呼吸系统症状，药物使用），生活质量（慢阻肺评估测试（CAT））。

6）诊断性肺量计检查：采用诊断性肺量计测量使用支气管扩张剂后的肺功能（由不同于测量呼气峰流速和微型肺量计的研究人员执行）。

上述过程由 2 名研究人员执行。

For peer review only - http://bmjopen.bmj.com/site/about/guidelines.xhtml

5. 本项研究会持续多久？

本研究将持续 1 年。

6. 参加本项研究的风险是什么？

根据研究需要可能会多次吹气，这可能会给一些受试者带来不适，如出现任何不适，请您随时告知研究人员。进行诊断性肺功能检查前我们会给予您沙丁胺醇，存在非常小的药物过敏的风险。如您在本研究中使用沙丁胺醇有不良反应，请务必告知研究人员。沙丁胺醇的副作用很少，可能会出现感觉摇晃，心率加快或头痛。上述情况可能在使用沙丁胺醇后立即出现，并在停用沙丁胺醇几分钟后消失。

问卷中的某些问题可能会让您感到不舒服，您可以拒绝回答。

7. 参加本项研究的获益是什么？

您不会因参加本项研究有直接获益，您的参与有助于我们发现有效的、具有经济效益的慢阻肺筛查方式，从而及早地发现慢阻肺患者，及时治疗，减轻他们的病情。

研究结束后，研究人员将对您解释肺功能检查结果，并对您的有关肺功能结果的问题进行解答。

8. 是否一定要参加并完成本项研究？

您是否参加这个研究完全是自愿的。如果您不愿意，可以拒绝参加，这对您目前或未来的医疗不会有任何负面影响。即使您同意参加以后，您也可以在任何时间改变主意，告诉研究者退出研究，您的退出不会影响您获得正常的医疗服务。

当您决定退出本项研究后，我们将停止收集您与本项研究有关的新数据。

9. 参加该项研究的费用和补偿

参与本项研究您不需要支付任何费用。

For peer review only - http://bmjopen.bmj.com/site/about/guidelines.xhtml

为了感谢您的参与和支持，我们会赠予一些小礼物，如洗衣粉、牙膏、洗手液等物品。

本项研究不提供交通费、误工费等金钱的报酬。

10. 发生研究相关伤害的处理？

本项研究不存在相关侵入性检查。

如果您对在上述研究过程中有任何不适，请及时告知现场的研究人员，我们会及时采取必要的医疗措施进行救治，或告知研究者（研究者联系人 潘子涵，电话 18701291196）。如经确认健康状况因参加本项研究而受到伤害时，将依照国家现行法律给予赔偿。

11. 我的信息会保密吗？

如果您决定参加本项研究，您参加研究及在研究中的个人资料均属保密。可以识别您身份的信息将不会透露给研究成员以外的人员，除非获得您的许可。所有的研究成员都被要求对您的身份保密。您的档案将保存在有锁的档案柜中，仅供研究人员查阅。为确保研究按照规定进行，必要时，政府管理部门或伦理委员会的成员按规定可以在研究单位查阅您的个人资料。

12. 数据存储

数据将被安全地存储于各社区卫生服务中心/卫生院，以及协调研究中心和英国伯明翰大学服务器上的在线数据库中。

欲了解更多信息，请访问：https://www.birmingham.ac.uk/breathewell。

13. 研究结果的发布

当我们分析完研究结果后，我们会将它们发布在学术期刊上。所有出版物将公布于 Breathe well 网站上。您不会在任何出版物中被识别出来。

14. 如果有问题或困难，该与谁联系？

For peer review only - http://bmjopen.bmj.com/site/about/guidelines.xhtml

如果您有与本项研究相关的任何问题，请联系 潘子涵医师，联系电话 18701291196。

如果您有与受试者自身权益相关的问题，可与北京大学第一医院生物医学研究伦理委员会联系，联系电话：010-66119025。

15. 经费资助

该研究由英国国家卫生研究院使用官方发展援助（ODA）资金委托进行。本研究中所表达的观点仅代表作者的观点，不代表 NHS，NIHR 或英国卫生部的观点。

16. 签字

研究编号 ID：

受试者声明

研究者向我说明了"慢性阻塞性肺疾病不同筛查策略在中国四个城市≥40 岁人群中的有效性及成本-效益的评价性研究"研究项目的背景、目的、步骤及参与研究的风险及获益事项，我有足够的时间和机会提出问题，研究者做出的解答我很满意。我知道当我有问题或想进一步获得信息应当与谁联系。我阅读了这份知情同意书，决定参加本项研究。我知道我可以在研究期间的任何时候无需任何理由都可以退出本项研究。我被告知我将得到这份知情同意书的副本，上面包含我和研究者的签名。

我允许研究人员在未来的相关研究中联系我，包括 Breathe Well 项目的其它研究。 ☐

受试者签名： 日期：

法定代理人签字【如适用】： 日期：

与受试者关系：

For peer review only - http://bmjopen.bmj.com/site/about/guidelines.xhtml

版本号：6.0 版本日期：2018.10.25

BUILDING RESEARCH ACROSS THE WORLD IN LUNG DISEASE

研究者声明

我已向研究对象（和法定代理人）讲解了“慢性阻塞性肺疾病不同筛查策略在中国四个城市≥40 岁人群中的有效性及成本-效益的评价性研究”的背景、目的、步骤及参与研究的风险及获益情况，给予他/她足够的时间阅读知情同意书、与他人讨论，并解答了其提出的有关本项研究的问题；我告诉了该研究对象遇到与研究相关的问题时的联系人方式；我告诉了该研究对象（或法定代理人）他/她可以在研究期间的任何时候无需任何理由退出本项研究。

研究者签名： 日期：

For peer review only

For peer review only - http://bmjopen.bmj.com/site/about/guidelines.xhtml

Evaluating screening strategies for identifying undiagnosed COPD in China: a Breathe Well project

Informed consent

We are conducting a study to evaluate the accuracy of different screening methods for chronic obstructive pulmonary disease（COPD）in people aged 40 years and older. We are inviting you to participate in this study because your condition meets the criteria for the enrolment. This sheet will give you a brief introduction for the purpose, process, benefits, and risks of this study. Please read it carefully before deciding whether you are interested in taking part, and you are welcome to discuss it with your family and friends. When the researcher explains and discusses the informed consent form, you can ask questions and ask him/her to explain anything to you if you have anything you don't understand. You can make a decision after discussing with your family, friends, and your doctor.

The study is funded through a collaboration with the University of Birmingham in the UK The primary investigator of this study is Chi Chunhua, chief physician of Peking University First Hospital.

1. Why do we conduct this study?

COPD has brought a huge economic burden to the country and has also brought about psychological and economic hardships for patients. China has a large population of patients with COPD. However, it faces the problems of high rate of missed and late diagnosis. The community is an important checkpoint for the prevention and treatment of chronic non-communicable diseases, and the community has unique advantages in early detection and early intervention in the COPD population. However, at present, what is cost-effective screening method for early detection of COPD patients is not clear. In view of this, we designed this study to assess the best screening methods for COPD in the community in China. The test commonly used to assess people's lung health is called spirometry, and this will be compared against screening tests including a peak flow meter, microspirometry and questionnaires.

2. Who will be invited to participate in this study?

For peer review only - http://bmjopen.bmj.com/site/about/guidelines.xhtml

Eligibility criteria:

Inclusion

- Aged ≥40 years
- Residing in the catchment areas

Exclusion

- Unable to do spirometry (e.g. dementia, lack of teeth-cannot make a good seal)
- Contraindicated for spirometry (chest infection in the last 3 weeks, coughing up blood in the last month, severe angina, uncontrolled high blood pressure, pneumothorax or history in the last 3 months of tuberculosis, heart attack, detached retina, or surgery on chest/abdomen/brain/ears/eyes
- Currently pregnant/breastfeeding
- Previous adverse reaction to Salbutamol

In addition, the researcher will judge according to your actual situation whether you are suitable for this study.

3. How many people will be recruited in this study?

We are recruiting approximately 2,000 subjects in the study, each community health centres need to recruit nearly 250 subjects.

4. What is the process of the study?

This study is a multi-centre cross-sectional survey (diagnostic test accuracy study). The research sites are Beijing (North), Chengdu (Southwest), Guangzhou (South), and Shenyang (Northeast). Each city selects two research sites: a community health service centre in urban area, and another in rural area.

Instruments model and drug information:

Instruments:

1）Expiratory peak flow meter(PEF): USPE

2）Microspirometer: COPD-6

3) Spirometer: ndd Easy On-PC (ndd Medizintechnik AG)

Funded by NIHR

Drug:

Bronchodilator: Salbutamol Aerosol (GSK)

The instruments and drugs used in this study have been approved by the State Food and Drug Administration (CFDA).

Study assessment visit:

If you participate in the study, you would attend one study assessment visit lasting approximately 90 minutes in total. If you are eligible for the study you will have your height and weight measured, before being asked to do three blowing tests and complete study questionnaires.

The data collection process is as flows:

Assessment station 1:

The researcher will confirm your eligibility for the study based on the criteria mentioned above and check for current chest infection (those with acute infections will be rebooked). If you are eligible you will then have opportunity to ask any questions about the study before completing the consent form with the researcher. As well as consenting to the main study, you will be asked to consent to being contacted about future research related to the study. Future contact will be optional and will not affect your ability to take part in the study.

Assessment station 2:

The researcher will measure your height and weight.

Assessment station 3:

You will be asked to perform blowing tests on two different devices, one called a peak flow meter and one called a microspirometer. You will be asked to perform 3 blows on each device, and further details are given below.

Lung function (peak flow)

For peer review only - http://bmjopen.bmj.com/site/about/guidelines.xhtml

A trained researcher will assess peak expiratory flow using a simple peak flow meter (USPE). For the main analysis, peak expiratory flow rates (PEFR) of <350 l/min for men and <250 l/min for women will be used to indicate a positive test Standardized researcher training will detail how to perform the technique and how to use the device. Each participant will perform three blows without administration of salbutamol, after which the researcher will record the highest PEF.

Lung function (microspirometry)

Microspirometry will be performed with minimal coaching by a trained researcher using a simple handheld microspirometer (COPD6), to measure FEV_1, FEV_6 and FEV_1/FEV_6 ratio. Microspirometer devices will be checked for calibration errors at the start of the study by the researchers. Standardized researcher training will detail how to perform the technique and how to use the device. Each participant will perform three blows using the device, after which the researcher will record the highest FEV_1 and FEV_6 values and the FEV_1/FEV_6 ratio. For the main analysis, FEV1/FEV6 ratios of <0.75 and <0.78 will be used to indicate a positive test.

Assessment station 4:

You will receive 400 µg of salbutamol using a large volume spacer, and then wait approximately 20 minutes before performing another blowing test.

Assessment station 5:

During the 20 minutes waiting period, you will be asked to complete study questionnaires by yourself, in a separate area of the assessment clinic, and return the completed forms to the researcher. The study pack will include four screening questionnaires (CAPTURE, CDQ, COPD-SQ and a symptom-based questionnaire). In addition to the screening questionnaires, the study pack will also include items relating to the following topics: demographic data (sex, age, marital status, education level, deprivation); lifestyle (smoking status, exercise); exposures (biomass smoke,

Funded by NIHR

For peer review only - http://bmjopen.bmj.com/site/about/guidelines.xhtml

occupational exposure to chemicals and particulates); health (medical diagnoses, comorbidities, respiratory symptoms, use of medications); quality of life (COPD assessment test (CAT)).

A member of the research team will be available to help you complete questionnaires if necessary.

Assessment station 6:

After completing the questionnaires, you will be asked to perform another blowing test using a portable spirometer (ndd Easy On-PC) that will be linked to a laptop. You may be asked to repeat this blowing test up to a maximum of 6 times.

5. How long will this study last?

This study will last for approximately one year.

6. What are the risks of participating in this study?

Blowing tests will be performed multiple times depending on the research needs. Multiple examinations may cause discomfort to some patients. If any discomfort occurs, please inform the researchers at any time. We will give you Salbutamol before the final blowing test. There is a very small risk of drug allergy. If you have adverse reactions with salbutamol in this study, please be sure to inform the investigator. Possible side effects from Salbutamol are rare and include feeling shaky, rapid heart rate or headache. If you experience any of these, it will be immediately after taking Salbutamol and will disappear after several minutes

Some questions in the questionnaire may make you feel uncomfortable, if so， ,you can refuse to answer.

7. What are the benefits of participating in this study?

You will not benefit directly from participating in this study. Your participation will help us find an effective, cost-effective method of screening for COPD, so that patients with COPD can be detected early, and they can be treated promptly to relieve their condition.

Funded by NIHR

At the end of the study assessment, researchers will explain your lung function results to you and answer any immediate questions you may have.

8. Is it necessary to participate in and complete this study?

It is entirely voluntary whether you participate in this research. If you do not want to, you can refuse to participate, which will not have any negative impact on your current or future medical care. Even if you agree to participate, you can change your mind at any time and tell the researchers to withdraw from the study. Your withdrawal will not affect your access to normal medical services.

If you decide to withdraw from this study, we will stop collecting new data related to this study from you.

9. Fees and compensation for participating in the study

You don't need to pay any cost to participate in this study.

We will compensate you for your time and support for participation by giving you small household tokens (e.g. washing powder, toothpaste, liquid soap).

This study does not provide monetary compensation such as transportation fees and losing of working time.

10. What happened to research-related injuries?

There are no relevant invasive tests in this study.

If you have any discomfort during the research process, please inform the researchers. We will promptly take the necessary medical measures for treatment or inform the researcher (main researcher **Pan Zihan**, Tel. **18701291196**). If it is confirmed that the health status is harmed because of participating in this study, you'll be compensated according to the current laws.

11. Is my information confidential?

If you decide to participate in this study, your participation in the study and personal data are confidential. Information that identifies you will not be disclosed to anyone other than the research member unless we have your permission. All research members are required to keep your identity confidential. Your file will be kept in a

Funded by NIHR

For peer review only - http://bmjopen.bmj.com/site/about/guidelines.xhtml

locked file cabinet for research purposes only. In order to ensure that the research is conducted in accordance with the regulations, members of the government management department or ethics committee may, as required, consult your personal data in the research sites.

12. Data storage

Data will be stored securely at the community centre, the co-ordinating study centre and on an online database that is held on servers at the University of Birmingham, UK. For further information, please refer to the Breathe Well website (https://www.birmingham.ac.uk/breathewell).

13. Publication of study findings

When we have analysed the results we will publish them in an academic journal. All publications will be available on the Breathe Well website. You will not be identified in any publication.

14. Who should I contact if I have problems or difficulties?

If you have any problems or difficulties related to this study, please contact **Dr. Pan Zihan**, her telephone number is **18701291196**.

If you have any problems or difficulties related to the subject's own rights, you can contact the Biomedical Research Ethics Committee of the **Peking University First Hospital** ,Tel: **010-66119025**.

15. Funding

The research was commissioned by the National Institute for Health Research using Official Development Assistance (ODA) funding. The views expressed are those of the author(s) and not necessarily those of the NHS, the NIHR or the Department of Health.

16. Signature

Study ID: ________________

Subject statement

The investigators explained to me the background, purpose, process, risks, and benefits of participating in ”a study to evaluate the accuracy of different screening methods for chronic obstructive pulmonary disease（COPD）in people aged 40 years and older”. I have enough time and opportunity to ask questions. I am very satisfied with the answers provided by the researchers. I know who I should contact when I have questions or want to get further information. I read this informed consent and decided to participate in this study. I know that I can withdraw from this study at any time during the study without any reason. I was told I would get a copy of this informed consent, which contains the signature of me and the researcher.

I give my permission to be contacted in the future for related research purposes including other studies in the Breathe Well programme. ☐

Subject's signature: Date:

Legal representative's signature [if applicable]: Date:

Relationship with the subject:

Researcher's statement

I have explained to the subjects (and legal representatives) the background, purpose, process, risks, and benefits of participating in “a study to evaluate the accuracy of different screening methods for chronic obstructive pulmonary disease（COPD）in people aged 40 years and older”. He/she had enough time to read the informed consent form, discuss it with others, and we answered his/her questions about the study; I told the subject how to contact the persons when they have research-related questions; I told him/her (or legal representative) that he/she may withdraw this study without any reason at any time during the study.

Researcher's signature: Date:

Funded by NIHR

北京大学第一医院生物医学研究伦理委员会审查批件

伦理审查编号：（2018）科研第（141）号-修正案　　EC 存档档案号：2018 研 141

伦理委员会批准日期：2018 年 11 月 07 日　批件有效期至：2019 年 11 月 06 日　定期跟踪审查频率：12 个月

项目名称	慢性阻塞性肺疾病不同筛查策略在中国四个城市≥40 岁人群中的有效性及成本-效益的评价性研究-横断面研究		
CFDA 批件号	/		
申办者	北京大学第一医院　健康管理中心		
临床研究科室	健康管理中心	主要研究者	迟春花
批准的文件	1、研究方案（版本号：6　版本日期：2018.10.25）<br>2、知情同意书（版本号：6.0　版本日期：2018.10.25）<br>3、研究对象信息表（版本号:v1　版本日期：2018.10.19）<br>4、肺部健康问卷（版本号：1.0　版本日期：2018.5.9）<br>5、调查问卷（版本号：v7　版本日期：2018.10.4）<br>6、中国案例报告表（版本号：Version3　版本日期：2018.10.25）		
本伦理委员会的人员组成和工作程序符合中国 GCP 以及国家相关规定			
伦理审查方式：■快速审查　审查时间：2018 年 11 月 07 日			
审查委员	张宝娓　谢鹏雁		
审查意见	同意按照上述批准的文件进行该临床试验。		

注意事项：

1. 本项临床试验应当在伦理委员会同意进行之日起 1 年内实施。逾期未实施的，本审查批件自行废止。
2. 研究应遵循本伦理委员会批准的方案执行，须符合 GCP 和《赫尔辛基宣言》的原则。
3. 自同意研究之日起，每隔 12 个月伦理委员会的定期跟踪审查（审查频度可能根据实际进展情况改变）；请在定期跟踪审查到期前 1 个月递交《定期跟踪审查表》。
4. 研究过程中，对研究方案和知情同意书等相关文件所作的任何修改，请交《修正案申请表》及“送审文件清单”中规定相关资料，并得到伦理委员会审查同意该修正后方可实施。
5. 发生严重不良事件或影响研究风险受益比的非预期不良事件，在向 CFDA 上报的同时向伦理委员会作书面通报，可以使用 CFDA 的《严重不良事件报告表》或本伦理委员会公布的《严重不良事件/非预期不良事件报告表》或其他有相关内容的报告表，但外文的报告需要有中文摘要。伦理委员会有权根据对其评估做出新的决定。
6. 不依从或违反方案应及时提交《不依从或违反方案报告表》。
7. 提前终止研究应及时提交《研究方案提前终止报告表》。
8. 研究完成后提交《研究结题报告表》和临床试验总结报告。
9. 及时书面报告其他伦理委员会的重要决定。

主任委员或副主任委员签名：[illegible]

北京大学第一医院生物医学研究伦理委员会（盖章）

2018 年 11 月 7 日

伦理委员会地址：北京市西城区大红罗厂街 6 号　邮编：100034　联系电话：010-66119025

215x296mm (200 x 200 DPI)

发件人: Susan Cottam <s.l.cottam@bham.ac.uk>
发送时间: 2018年10月22日星期一 17:30
收件人: Andy Dickens
主题: Application for Ethical Review ERN_18-1177

Dear Dr Dickens

Re: "A study to evaluate the effectiveness and cost-effectiveness of different screening strategies for identifying undiagnosed COPD in China, amongst residents (≥ 40 years) in four cities"
Application for Ethical Review ERN_18-1177

Thank you for your application for ethical review for the above project, which was reviewed by the Science, Technology, Engineering and Mathematics Ethical Review Committee.

On behalf of the Committee, I confirm that this study now has full ethical approval.

I would like to remind you that any substantive changes to the nature of the study as described in the Application for Ethical Review, and/or any adverse events occurring during the study should be promptly bought to the Committee's attention by the Principal Investigator and may necessitate further ethical review.

Please also ensure that the relevant requirements within the University's Code of Practice for Research and the information and guidance provided on the University's ethics webpages (available at https://intranet.birmingham.ac.uk/finance/accounting/Research-Support-Group/Research-Ethics/Links-and-Resources.aspx) are adhered to and referred to in any future applications for ethical review. It is now a requirement on the revised application form (https://intranet.birmingham.ac.uk/finance/accounting/Research-Support-Group/Research-Ethics/Ethical-Review-Forms.aspx) to confirm that this guidance has been consulted and is understood, and that it has been taken into account when completing your application for ethical review.

Please be aware that whilst Health and Safety (H&S) issues may be considered during the ethical review process, you are still required to follow the University's guidance on H&S and to ensure that H&S risk assessments have been carried out as appropriate. For further information about this, please contact your School H&S representative or the University's H&S Unit at healthandsafety@contacts.bham.ac.uk.

Kind regards

Susan Cottam
Research Ethics Officer
Research Support Group
C Block Dome
Aston Webb Building
University of Birmingham
Edgbaston B15 2TT
Tel: 0121 414 8825
Email: s.l.cottam@bham.ac.uk
Web: https://intranet.birmingham.ac.uk/finance/RSS/Research-Support-Group/Research-Ethics/index.aspx

Please remember to submit a new Self-Assessment Form for each new project.

You can also email our team mailbox ethics-queries@contacts.bham.ac.uk with any queries relating to the University's ethics process.

For peer review only - http://bmjopen.bmj.com/site/about/guidelines.xhtml

Click Research Governance for further details regarding the University's Research Governance and Clinical Trials Insurance processes, or email researchgovernance@contacts.bham.ac.uk with any queries relating to research governance.

Notice of Confidentiality:
The contents of this email may be privileged and are confidential. It may not be disclosed to or used by anyone other than the addressee, nor copied in any way. If received in error please notify the sender and then delete it from your system. Should you communicate with me by email, you consent to the University of Birmingham monitoring and reading any such correspondence.

For peer review only

BMJ Open

A study to evaluate the effectiveness and cost-effectiveness of different screening strategies for identifying undiagnosed COPD amongst residents (≥40 years) in four cities in China: protocol for a multicenter cross-sectional study. On behalf of the Breathe Well group.

Journal:	*BMJ Open*
Manuscript ID	bmjopen-2019-035738.R1
Article Type:	Protocol
Date Submitted by the Author:	14-Mar-2020

Complete List of Authors:	Pan, Zihan; Peking University First Hospital, ; Peking University Health Science Centre,<br>Dickens, Andrew; University of Birmingham, Institute of Applied Health Research<br>Chi, chunhua; Peking University First Hospital, General Practice<br>Kong, Xia; Peking University First Hospital, General Practice<br>Enocson, Alexandra; University of Birmingham, Applied Health Research<br>Adab, Peymane; University of Birmingham Institute of Applied Health Research, Institute of Applied Health Research<br>Cheng, Kar Keung; University of Birmingham Institute of Applied Health Research<br>Sitch, Alice; University of Birmingham Institute of Applied Health Research<br>Jowett, Sue; University of Birmingham Institute of Applied Health Research<br>Jordan, Rachel; University of Birmingham Institute of Applied Health Research

**Primary Subject Heading**:	Diagnostics
Secondary Subject Heading:	Respiratory medicine
Keywords:	Chronic airways disease < THORACIC MEDICINE, HEALTH ECONOMICS, PUBLIC HEALTH, PRIMARY CARE

SCHOLARONE™
Manuscripts

I, the Submitting Author has the right to grant and does grant on behalf of all authors of the Work (as defined in the below author licence), an exclusive licence and/or a non-exclusive licence for contributions from authors who are: i) UK Crown employees; ii) where BMJ has agreed a CC-BY licence shall apply, and/or iii) in accordance with the terms applicable for US Federal Government officers or employees acting as part of their official duties; on a worldwide, perpetual, irrevocable, royalty-free basis to BMJ Publishing Group Ltd ("BMJ") its licensees and where the relevant Journal is co-owned by BMJ to the co-owners of the Journal, to publish the Work in this journal and any other BMJ products and to exploit all rights, as set out in our licence.

The Submitting Author accepts and understands that any supply made under these terms is made by BMJ to the Submitting Author unless you are acting as an employee on behalf of your employer or a postgraduate student of an affiliated institution which is paying any applicable article publishing charge ("APC") for Open Access articles. Where the Submitting Author wishes to make the Work available on an Open Access basis (and intends to pay the relevant APC), the terms of reuse of such Open Access shall be governed by a Creative Commons licence – details of these licences and which Creative Commons licence will apply to this Work are set out in our licence referred to above.

Other than as permitted in any relevant BMJ Author's Self Archiving Policies, I confirm this Work has not been accepted for publication elsewhere, is not being considered for publication elsewhere and does not duplicate material already published. I confirm all authors consent to publication of this Work and authorise the granting of this licence.

For peer review only

A study to evaluate the effectiveness and cost-effectiveness of different screening strategies for identifying undiagnosed COPD amongst residents (≥40 years) in four cities in China: protocol for a multicenter cross-sectional study. On behalf of the Breathe Well group.

Zihan Pan[1]; Andrew P Dickens[2]; *Chunhua Chi[1]; Xia Kong[1]; Alexandra Enocson[2]; Peymané Adab[2]; Kar Keung Cheng[2]; Alice Sitch[2,3]; Sue Jowett[2], Rachel E Jordan[2] on behalf of the Breathe Well Group.

Corresponding author:
Name: Chunhua Chi
Postal address: Peking University First Hospital, No.8 XiShiKu Street, Xicheng District, Beijing, 100034, China
E-mail: chichunhua@vip.163.com,
Telephone: +86 13910987530,
Fax numbers: +86 010-66158996

Co-authors details:
1. Department of General Practice, Peking University First Hospital, Beijing, China
2. Institute of Applied Health Research, University of Birmingham, Birmingham, UK.
3. NIHR Birmingham Biomedical Research Centre, University Hospitals Birmingham NHS Foundation Trust and University of Birmingham, UK

Key word: COPD; diagnostic accuracy test; screening strategies, health economics; primary care

Word count: 3952 words

For peer review only

Abstract

Introduction: The latest COPD epidemiology survey in China estimated that there were 99 million potential COPD patients in the country, the majority of whom are undiagnosed. Screening for COPD in primary care settings is of vital importance for China, but it is not known which strategy would be the most suitable for adoption in primary care. Studies have been conducted to test the accuracy of questionnaires, expiratory peak flow meters, and microspirometers to screen for COPD, but no studies have directly evaluated and compared the effectiveness and cost-effectiveness of these methods in the Chinese setting.

Methods and analysis: We present the protocol for a multicenter cross-sectional study, to be conducted in 8 community hospitals from 4 cities amongst Chinese adults aged 40 years or older to investigate the effectiveness and cost-effectiveness of different case finding methods for COPD, and determine the test performance of individual and combinations of screening tests and strategies in comparison with quality diagnostic spirometry. Index tests are screening questionnaires (CDQ, CAPTURE, symptom-based questionnaire, COPD-SQ), microspirometer and peak flow. Each participant will complete all of these tests in one assessment. The primary analysis will compare the performance of a screening questionnaire with a handheld device. Secondary analyses will include the comparative performance of each index test, as well as a comparison of strategies where we use a screening questionnaire and a handheld device. Approximately 2000 participants will be recruited over 9-12 months.

Ethics and dissemination: The study has been approved by Peking University Hospital and University of Birmingham. All study participants will provide written informed consent. Study results will be published in appropriate journal and presented at national and international conferences, as well as relevant social media and various community/stakeholder engagement activities.

Trial registration: ISRCTN13357135.

Keywords: COPD; diagnostic accuracy test; screening strategies, health economics; primary care

For peer review only

Strengths and limitations of the study

- This is the first study to compare the effectiveness and cost-effectiveness of selected screening tests (questionnaires, peak flow meter and microspirometer) and strategies to screen for COPD in China.
- Recruiting participants from both urban and rural community hospitals will maximize the generalisability to primary care patients
- Including four different screening questionnaires enables comparison of their test performance within a Chinese COPD population
- Using blinded researchers to administer quality diagnostic spirometry minimizes the risk of reviewer bias.
- The study will be conducted in four cities across China, which are geographically disparate but may not be representative of China as a whole.

Introduction

Chronic obstructive pulmonary disease (COPD) is a common, preventable and treatable chronic condition characterized by persistent respiratory symptoms and airflow limitation[1]. Despite COPD being the 3rd leading cause of death in the world[2], COPD is underdiagnosed throughout the world due to multiple reasons including low awareness of the disease and its consequences amongst the public and primary care health professionals, and the low use of spirometry[3]. While studies report the prevalence of undiagnosed COPD as being approximately 70% in Spain[3] and Poland[4] amongst those with the condition, a recent study in China reported that 96% of those with spirometry-confirmed COPD did not have a diagnosis [5]. Data from the United States National Health and Nutrition Examination Survey (NHANES) revealed those with undiagnosed COPD were characterized by fewer symptoms [6], this is reflected in China where 68% of undiagnosed people were asymptomatic[7]. What's more, about 30% of COPD patients were asymptomatic, those people were more likely to be underdiagnosed[4,8].

The Global Initiative for Chronic Obstructive Lung Disease(GOLD) define individuals as being at high risk of COPD if they have chronic respiratory symptoms, exposure to risk factors, or medical/family history of respiratory disease [1]. According to the above definition, about 90% of people aged ≥40 years in China were at high risk of COPD in 2014[9]. The prevalence of diagnosed COPD in China was 13.7% in 2015[5]. Considering the substantial proportion of the Chinese population that is at risk of undiagnosed disease, screening for COPD in China is essential. Recently, China has called for national policies and programmes for the prevention and early detection of COPD[5,10]. In line with this, government agencies have recommended the incorporation of pulmonary function tests into routine health examinations in China's 13th Five-Year Plan for Health care[11].

Whilst guidelines recommend that COPD is diagnosed based on spirometry and symptomology[1], spirometry is not always available in primary care settings in China [12,13]. Among a large population of COPD patients in China, less than 12% had ever been tested using spirometry[5,10]. As a result, there is a need for simple and affordable COPD screening tools in primary care settings.

While COPD screening programmes are not currently recommended by the United States[14] or United Kingdom[15] due to insufficient evidence of health benefits, the national policy do

For peer review only

recommend screening for undiagnosed COPD in China[11]. Despite the support for screening in China, there is no recommendation on the best strategy or approach to use. Multiple screening questionnaires have been developed to identify patients at risk of COPD, either in primary or secondary care settings[16-20]. Questionnaire items include the presence of respiratory symptoms (e.g. wheeze, dyspnea, cough) while some tools also explore exposures, smoking history and age. The questionnaires are all designed to be self-completed, but vary regarding the populations in which they were developed/validated e.g. general population or targeted groups such as symptomatic patients, current smokers etc. Microspirometers are small handheld devices that measure lung function, which are low cost, quick to use and require minimal coaching for patients. Peak flow monitors are simple, low cost devices that measure how much air patients can expel during a forced expiration (peak expiratory flow, PEF), and evidence indicates that these devices may also be suitable as a possible screening tool for COPD[18,21,22].

Screening tests can be used individually or in combination as screening 'strategies'. Systematic reviews and more recent primary studies typically assess the use of a single test, concluding that many of the available tests may be appropriate for use in COPD screening[23,24,25]. However, studies in community settings in China are limited and it is not known which screening test or strategy would be most appropriate to use. As a middle-income country with a large potential COPD population, it is important to explore the most effective and cost-effective screening strategy. Accurately detecting individuals who merit referral for quality diagnostic spirometry could minimize the number of ineligible referrals, thus protecting health system resources and ensuring appropriate and timely treatment for those subsequently diagnosed.

Aims and objectives

Aim

The aim of the study is to identify the most effective and cost effective screening strategy for identifying undiagnosed COPD amongst those aged 40 years or older in China.

Objectives

- To determine the comparative test performance of all screening tests and strategies in diagnosing COPD (confirmed by quality diagnostic spirometry).
- To evaluate the cost-effectiveness of each screening strategy.

Methods and analysis

Study recruitment commenced in February 2019 and ended in December 2019.

Design

Multicenter cross-sectional test accuracy study. The study is registered at *http://www.isrctn.com* (ISRCTN13357135).

The STARD guideline[26] was used for reporting studies of diagnostic test accuracy to inform the content of the protocol and we will use this to report the study.

Study setting

The study will be implemented in four cities in China: Beijing (North), Chengdu (Southwest),

For peer review only

Guangzhou (South), Shenyang (Northeast). Cities were purposively selected to represent urban/rural settings and differing geographic areas of the country, where exposures, lifestyles and the prevalence of COPD may differ. The national study about COPD prevalence in 2007 in China was taken as a selection reference. Each selected city had the highest prevalence of COPD in each geographic area, prevalence is shown on the map[27]. Participants will be recruited from eight community health service centers (CHSC); 1 rural and 1 urban in each city. The study sites are shown on the map (Figure 1).

Study population

Inclusion criteria

- Aged ≥40 years
- Residing in the catchment areas of the participating CHSCs in the four cities

Exclusion criteria

- Unable to perform spirometry (e.g. dementia, lack of teeth-cannot make a good seal)
- Contraindicated for spirometry (chest infection in the last 3 weeks, coughing up blood in the last month, severe angina, uncontrolled high blood pressure, pneumothorax or history in the last 3 months of tuberculosis, heart attack, detached retina, or surgery on chest/abdomen/brain/ears/eyes
- Currently pregnant/breastfeeding
- Previous adverse reaction to Salbutamol

Recruitment

Participants will be recruited to the study via two main routes; advertisement or doctor referral. Participating CHSCs and their satellite offices will advertise the study by displaying posters and sending messages to their secure/closed/other resident WeChat social media groups, inviting residents to contact the research team if they are interested in taking part. Potentially eligible patients visiting the participating CHSCs will be given a study information sheet by the healthcare professionals and invited to attend a study assessment with researchers. Study participants will also be encouraged to promote the study to their family members and friends. The recruitment route of all participants will be recorded.

For the first 4 weeks of recruitment, the study will only be conducted in Beijing to allow all study processes to be piloted and altered as required, after which it will be implemented in the other 3 cities. Recruitment flow through the study is summarized in Figure 2.

Study tests

The study will use a paired design, with all participants receiving the index tests and reference test during the same study assessment. The study will administer a total of 6 index tests (pre-bronchodilator peak flow and micro spirometry, and 4 screening questionnaires) and one reference test (post-bronchodilator quality diagnostic spirometry) to each participant.

Index tests

- Lung function test—Peak flow

For peer review only

A trained researcher will assess peak expiratory flow using a simple peak flow meter (USPE, China). Each participant will perform three blows without administration of bronchodilator, after which the researcher will record the highest PEF. For the main analysis, peak expiratory flow rates (PEFR) of <350 l/min for men and <250 l/min for women will be used to indicate a positive test [18].

- Lung function test—Microspirometer

Microspirometry will be performed with minimal coaching by a trained researcher using a simple handheld microspirometer (Vitalograph COPD6), to measure FEV_1, FEV_6 and FEV_1/FEV_6 ratio. Each participant will perform three blows using the device, after which the researcher will record the highest FEV_1 and FEV_6 values and the FEV_1/FEV_6 ratio. For the main analysis, FEV_1/FEV_6 ratios of <0.75[28] and <0.78[29] will be assessed to indicate a positive test.

- Screening questionnaires

Four screening questionnaires will be used in the study; the CDQ[17,30], the COPD-SQ[19], a symptom-based questionnaire[31], and CAPTURE[18](see Appendix 1). The selection of questionnaires maximizes symptoms being assessed and minimizes duplication of items, whilst allowing comparison of the most relevant questionnaires. Recommended cut-points for each questionnaire will be used to identify those at risk of COPD for diagnostic spirometry, with potential additional analyses to explore optimal cut-points.

Reference test

Post-bronchodilator quality diagnostic spirometry (20-60 minutes after administration of 400ug Salbutamol) will be performed by a trained researcher using a portable spirometer (ndd Easy On-PC). Lung function data including FEV_1, FVC and FEV_1/FVC ratio will be recorded in the ndd software, and will also be imported to the study REDCap database. Accuracy of the device flow heads will be verified at the start of each assessment day by the researchers; calibration is not required. Participants will perform a maximum of six blows, or less if repeatability within 100mls or 5% is achieved (ARTP standards (2013))[32]. For the purposes of this study, a COPD diagnosis will be defined as airflow obstruction based on the lower limit of normal using the Global Lung Initiative (GLI) equations, according to post-bronchodilator quality diagnostic spirometry.

Ordering of assessments

Index tests will be conducted before the reference test for all participants, and the reference test will be administered by a different researcher who will be blind to the previous test results. To decrease the potential training effect within the index tests, the order of the peak flow and microspirometer will be alternated i.e. approximately half of the participants will perform peak flow first and vice versa. The screening questionnaires will always be completed after administration of Salbutamol, during the 20-60 minutes timeframe permitted prior to the reference test. Due to use of pre-printed study material, the order of the screening questionnaires will not be alternated.

Besides that, participants' standing height (stadiometer) and weight (scales) will be measured. Participants will also be asked to complete a study questionnaire by themselves, in a separate area of the assessment clinic, and return the completed forms to the researcher. The study questionnaire (see Appendix 2) will include items relating to the following topics: demographic data (sex, age, marital status, education level, deprivation); smoking status; exposures (biomass smoke, occupational exposure to chemicals and particulates); health (medical diagnoses [inc.

For peer review only

COPD, asthma, TB etc], comorbidities, respiratory symptoms); quality of life (COPD assessment test (CAT)). A member of the research team will be available to help participants to complete questionnaires if necessary.

Data collection

Study assessment

The study assessment will last approximately 80 minutes, including 6 stations. There will be 2 researchers at each assessment clinic to enable the assessments to run in parallel, and all data will be recorded on CRFs(case report form), ensuring standardized data collection/recording.

At the end of the study assessment, researchers will provide all participants with information about the level of their airway obstruction, suggest they contact a doctor if appropriate, and answer any immediate questions they may have. The flow of participants is presented in figure 2.

Resource use data

To calculate the health care costs of delivering each screening strategy, we will determine the unit costs and quantity of any equipment, medication and consumables required, as well as staff type and grade, staff time taken to deliver each individual test and use of facilities. The staff time taken will be collected with a simple questionnaire for researchers to fill in for each test (see Appendix 3). Equipment costs (peak flow meters, spirometers) will be amortized over the estimated lifespan of the equipment. The cost per patient visit will be calculated using assumptions regarding the total number of patients the equipment will be used for. In addition, each individual test will be timed at a sample of assessment clinics so an overall mean time and range for each test can be estimated.

Statistical methods

Sample size

The Alonzo method for paired test accuracy studies[33] was used to calculate the sample size, assuming independence of tests and a prevalence of 12%, we will have 90% power to detect a difference in sensitivity of 10% (95% vs 85%[18,34,35,30]) with 1622 participants. If the sensitivity of tests is slightly lower in this population (90% vs 80%) we would have 90% power to detect this difference with a larger sample of 2279 participants.

Analysis plan

Data will be analysed using Stata v15.

Our primary analysis will compare the performance of a screening questionnaire (CAPTURE) with a handheld device (peak flow meter). Secondary analyses will include the comparative performance of each index test, as well as a comparison of strategies where we use a screening questionnaire and a handheld device.

The performance of each index test when diagnosing COPD (confirmed by quality diagnostic spirometry) will be investigated by presenting 2x2 tables and calculating the sensitivity, specificity, positive predictive value and negative predictive value, along with 95% confidence intervals. For the tests with a continuous score, receiver operator curve (ROC) analysis with AUC (with 95% CIs) will be produced. Comparisons of test accuracy between different index tests and different test strategies will be conducted using McNemar's test and logistic regression modelling.

Sensitivity analyses will explore the impact on test performance of the index tests and strategies

For peer review only

when using different definitions of COPD, including i) a combination of spirometry data and clinical confirmation, and ii) using the GOLD definition (fixed ratio (FEV_1/FVC <0.7) of airflow obstruction. Additional sensitivity analyses may explore the impact of spirometry quality as well as exploring optimal cut-points for the screening tests, in recognition that test performance will be dependent on the cut-points used.

A fully incremental cost-effectiveness analysis will be undertaken from a health care perspective to calculate the cost per true case detected for all pre-determined strategies. The strategies (including combinations) will be ordered by the number of true cases detected, from least to greatest, and the principles of dominance and extended dominance will be applied to eliminate redundant strategies from the analysis. Sensitivity analysis will be undertaken to explore the impact on results of any changes in assumptions, e.g. time taken for a strategy.

Training

A two-day training event will be organized for all researchers to ensure standardized study processes are followed at all research sites. Training will cover study processes and assessment techniques as well as expert teaching regarding respiratory physiology and spirometry lung function tests. Researchers' competency in conducting spirometry will be certified at the end of the training. Spirometry traces from practice sessions will be over-read by an expert to ensure sufficient quality prior to participant recruitment commences. Local respiratory specialists will over-read all spirometry tests during the study period, to ensure quality is maintained. During site initiation visits, the study team will observe a complete study assessment to ensure researchers adhere to the study protocol. The study will conduct monitoring site visits throughout the study period.

Patient and public involvement

The research team conducted a research prioritization exercise with patients, clinicians and policy makers, and the need to identify effective screening strategies for undiagnosed COPD was one of the research areas prioritized. All stakeholders involved in this exercise will receive study updates twice a year, will be kept informed of findings and will be consulted at the end of the study regarding implications for practice and policy decisions, as well as advice on appropriate dissemination of study findings.

A patient advisory group (PAG) has been set up, which is funded to meet at approximately quarterly intervals or according to need, and will advise on a range of aspects of the design, conduct, analysis and dissemination of the study. The PAG will discuss issues as requested by the CIs and the chair will report their comments back to the investigators.

In addition, the study has a Trial Steering Committee (TSC) that meets regularly and comprises various independent members, including a patient and a clinician representative as well as international experts in respiratory research. The TSC also includes several members of the study research team.

Ethics and dissemination

Ethics and informed consent

The study has been approved by Peking University First Hospital (2018-R-141, PUFH) (see Appendix 4) and University of Birmingham (ERN_18-1177, UoB) (see Appendix 5). Residents responding to

For peer review only

the study invitation will be given the study information sheet with enough time to read it and will have opportunity to ask the researcher any questions about the study. Interested respondents who are eligible for the study will be asked to sign a consent form (see Appendix 6), or if unable to consent, a family member will be asked to sign on their behalf. Consent will also be sought to allow the research team to contact participants about future studies related to the Breath Well program; this is optional and will not affect eligibility for the study described in this paper.

Indemnity

The study is not an intervention study, and as such poses low risk to participants. However, clinical insurance was purchased in case of Serious Adverse Events (SAEs).

Data storage

Study data will be entered into a bespoke REDCap online database. All electronic data held by the research team will be password protected and stored on encrypted study laptops. Paper-based data will be held in locked filing cabinets in the study office in each site. The research team will conduct monitoring visits of all research sites during the recruitment period to ensure data are being collected, entered and stored according to pre-specified study working instructions.

Dissemination and Publication policy

Study results will be published in peer-reviewed journals and presented at national and international conferences, as well as relevant community/stakeholder engagement activities. Participants who explicitly express a wish to be informed about the research outcome will be contacted and offered to receive an article or poster with a lay summary of the study.

Discussion

This study aims to identify the most effective and cost-effective screening strategies for identifying undiagnosed COPD in the primary care setting in China.

To the best of our knowledge, this is the first study to assess the accuracy of different COPD screening strategies including screening questionnaires, peak flow and microspirometer measurement. This study is being conducted in a range of community hospitals from rural and urban areas which are broadly representative of primary care institutions in China. The planned cost-effectiveness analysis will calculate the cost per true case detected for each strategy, which will help inform decisions about the future feasibility of screening strategies within the primary care setting in China. This trial should inform primary care across China and elsewhere with similar healthcare systems, and help to direct current effort towards case-finding more efficiently.

While the study will be conducted in four purposively selected cities, it is possible that additional cities will be required to obtain a representative sample of the Chinese COPD population. However, increasing the number of study locations would have introduced difficulties such as training and monitoring study sites, thus we believe the selected cities represent an acceptable balance between study feasibility and representativeness. Furthermore, estimates of effectiveness are based on measurements undertaken under research conditions. Whilst the screening tests are likely to be reproducible in routine practice, it is possible that peak flow and microspirometer measures could be done to a higher standard in research settings, leading to potential overestimation of effectiveness.

For peer review only - http://bmjopen.bmj.com/site/about/guidelines.xhtml

This study also helps building research capacity within primary care, as it is the first respiratory study for the participating community hospitals and the majority of General Practioner (GP) researchers being taught how to conduct high quality spirometry will have no prior experience and might have difficulty in understanding the research process.

Recent health policies have seen lung function testing being incorporated into a routine health examination programme amongst the general population , and objectives being set to increase the proportion of those over 40 years old received lung function tests from 7.1% in 2017 to 15% in 2020 and 25% in 2025[36]. Considering the increasing importance of lung function testing in China and the intensive spirometry training given to clinicians through this study, we believe this study could also help improve the quality of COPD management in primary care in China.

Considering that there is no "GP first contact" in China yet, it is challenging to plan how best to attract people attending community hospitals and recruit them into the study. However, voluntary pulmonary function screening identifies high rates of undiagnosed asymptomatic COPD[7]. How to encourage residents to volunteer to participate in screening is also something we need to consider. It is also hard to anticipate residents' willingness to participate in this study and how participants will respond to the study measures. However, what is worth mentioning, besides posters, referred by doctors, friends or family members, Wechat, a social media which has a prominence in Chinese society now, also plays an important role in the recruitment process to inform residents or disseminate the programme. Last but not least, it will be important to discuss how this approach can be rolled out from a trial setting into routine practice. Real world study may be the most appropriate method to make it clear how the validated screening strategy works in practices.

COPD screening is extremely important to China and its 99.9 million potential COPD patients[5]. This study will provide robust evidence about the effectiveness and cost-effectiveness of different COPD screening methods and strategies and confirm which the best COPD screening strategy is. The service might be a template for delivery of a procedural screening strategy that can reach large numbers of an under recognized population. Although the long-term benefits of screening are still to be proven, this programme has capacity to contribute significantly to improving public health.

Author affiliations

1. Department of General Practice, Peking University First Hospital, Beijing, China

2. Institute of Applied Health Research, University of Birmingham, Birmingham, UK.

3. NIHR Birmingham Biomedical Research Centre, University Hospitals Birmingham NHS Foundation Trust and University of Birmingham, UK

Contributors

Zihan Pan and Andrew P Dickens wrote the protocol paper with input from all other authors. Rachel E Jordan led the design of the trial, with contributions and advice from all other investigators. Chunhua Chi, Xia Kong, Peymane Adab, KK Cheng contributed to decisions on outcome measures. Chunhua Chi and KK Cheng advised on involving GP practices, Rachel E Jordan, Peymane Adab, Alexandra Enocson and Andrew P Dickens advised on lung function testing. Andrew P Dickens and Rachel E Jordan designed the intervention. Alice Sitch and Sue Jowett designed the analysis plan and economic evaluation. Chunhua Chi was the local PI. All authors have read and approved the final draft.

Funding

For peer review only

This research was funded by the National Institute for Health Research (NIHR) NIHR global group on global COPD in primary care, University of Birmingham, (project reference: 16/137/95) using UK aid from the UK Government to support global health research. The views expressed in this publication are those of the author(s) and not necessarily those of the NIHR or the UK Department of Health and Social Care.

Competing interests

The authors declare that they have no competing interests.

Ethics approval

Peking University First Hospital Ethics Committee (2018-R-141, PUFH) and the Science, Technology, Engineering and Mathematics Ethical Review Committee, University of Birmingham (ERN_18-1177, UoB).

Acknowledgements

The views expressed in this publication are those of the author(s) and not necessarily those of the NIHR or the Department of Health and Social Care. We gratefully acknowledge International Primary Care Respiratory Group (IPCRG) for introducing us to the primary care networks involved in this study and for its continued facilitation of clinical engagement. This paper presents independent research supported by the NIHR Birmingham Biomedical Research Centre at the University Hospitals Birmingham NHS Foundation Trust and the University of Birmingham.

Additional file

Additional file: Appendix 1. Screening questionnaires. **Appendix 2**. Study questionnaire. **Appendix 3**. Health economic questionnaire. **Appendix 4**. Ethic approvals of Peking University First Hospital **Appendix 5** Ethic approvals of University of Birmingham**.** **Appendix 6** Patient informed consent.

Figure legends

Figure 1 the map of Breathe Well-China research sites

Figure 2 flow of participants

For peer review only

References

[1] Global Initiative for Chronic Obstructive Lung Disease (GOLD).Global strategy for the diagnosis, management, and prevention of chronic obstructive pulmonary disease (2018 report).https://goldcopd.org/wp-content/uploads/2018/11/GOLD-2019-v1.7-FINAL-14Nov2018-WMS.pdf.

[2] Lozano R, Naghavi M, Foreman K, et al. Global and regional mortality from 235 causes of death for 20 age groups in 1990 and 2010: a systematic analysis for the Global Burden of Disease Study 2010. Lancet. 2012 Dec 15;380(9859):2095-128.

[3] López-Campos JL, Tan W, Soriano JB. Global burden of COPD. Respirology. 2016. 21(1): 14-23.

[4] Bednarek M, Maciejewski J, Wozniak M, Kuca P, Zielinski J. Prevalence, severity and underdiagnosis of COPD in the primary care setting. Thorax. 2008. 63(5): 402-7.

[5] Wang C, Xu J, Yang L et al. Prevalence and risk factors of chronic obstructive pulmonary disease in China (the China Pulmonary Health [CPH] study): a national cross-sectional study. Lancet. 2018 Apr 28;391(10131):1706-1717.

[6] Hangaard S, Kronborg T, Hejlesen OK. Characteristics of Subjects With Undiagnosed COPD Based on Post-Bronchodilator Spirometry Data. Respir Care. 2019. 64(1): 63-70.

[7] Wang S, Gong W, Tian Y. Voluntary pulmonary function screening identifies high rates of undiagnosed asymptomatic chronic obstructive pulmonary disease. Chron Respir Dis. 2016. 13(2): 137-43.

[8] Lu M, Yao WZ, Zhong NS,et al. Asymptomatic patients of chronic obstructive pulmonary disease in China. Chin Med J (Engl). 2010 Jun;123(12):1494-9.

[9] Bao HL, Cong S, Wang N, et al. Survey and analyses of population at high risk of chronic obstructive pulmonary disease in China,2014. Chin J Epidemiol, May 2018, Vol.39, No.5.(in Chinese)

[10] Fang L, Gao P, Bao H, et al. Chronic obstructive pulmonary disease in China: a nationwide prevalence study. Lancet Respir Med. 2018. 6(6): 421-430.

[11] National Health and Family Planning Commission of the People's Republic of China.The 13th Five-Year Plan for Healthcare. Dec 27, 2016. http://www.gov.cn/zhengce/content/2017-01/10/content_5158488.htm. (in Chinese).

[12] Wen FQ, Shen YC, Chen .Pulmonary function test in the management of chronic obstructive pulmonary disease patients in China: challenges and countermeasures. Chin J Tuberc Respir Dis, December 2017, Vol.40, No.12.(in Chinese)

[13] He QY. Standardized lung function measurement technology to improve the lung function determination. ChinJTubercRespirDis, December2006, Vol.29, No.12.(in Chinese)

[14] US Preventive Services Task Force. Screening for chronic obstructive pulmonary disease: US Preventive Services Task Force Recommendation Statement. JAMA 315, 1372–1377 (2016)

[15] UK National Screening Committee. An evaluation of screening for COPD against the National Screening Committee criteria (2013)

[16] Calverley PM, Nordyke RJ, Halbert RJ, Isonaka S, Nonikov D. Development of a population-based screening questionnaire for COPD. COPD. 2005. 2(2): 225-32.

[17] Price DB, Tinkelman DG, Halbert RJ, et al. Symptom-based questionnaire for identifying

COPD in smokers. Respiration. 2006. 73(3): 285-95.
[18] Martinez FJ, Mannino D, Leidy NK, et al. A New Approach for Identifying Patients with Undiagnosed Chronic Obstructive Pulmonary Disease. Am J Respir Crit Care Med. 2017 Mar 15;195(6):748-756.
[19] Zhou YM, Chen SY, Tian J,et al. Development and validation of a chronic obstructive pulmonary disease screening questionnaire in China. Int J Tuberc Lung Dis. 2013 Dec;17(12):1645-51.
[20] Martinez FJ, Raczek AE, Seifer FD, et al. Development and initial validation of a self-scored COPD Population Screener Questionnaire (COPD-PS). COPD. 2008. 5(2): 85-95.
[21] Jackson H, Hubbard R. Detecting chronic obstructive pulmonary disease using peak flow rate: cross sectional survey. BMJ. 2003. 327(7416): 653-4.
[22] Tian J, Zhou Y, Cui J, et al. Peak expiratory flow as a screening tool to detect airflow obstruction in a primary health care setting. Int J Tuberc Lung Dis. 2012. 16(5): 674-80.
[23] Haroon S, Jordan R, Takwoingi Y, Adab P. Diagnostic accuracy of screening tests for COPD: a systematic review and meta-analysis. BMJ Open. 2015. 5(10): e008133.
[24] Accuracy of Vitalograph lung monitor as a screening test for COPD in primary care. npj Primary Care Respiratory Medicine (2020) 30:2 ; https://doi.org/10.1038/s41533-019-0158-2
[25] Can CAPTUREbe used to identify undiagnosed patients with mild-to-moderate COPD likely to benefit from treatment? International Journal of COPD 2018:13 1901–1912.
[26] Bossuyt, P. M. et al. STARD 2015: an updated list of essential items for reporting diagnostic accuracy studies. BMJ 351, h5527, doi:10.1136/bmj.h5527 (2015).
[27] Zhong N, Wang C, Yao W, et al. Prevalence of chronic obstructive pulmonary disease in China: a large, population-based survey. Am J Respir Crit Care Med 2007; 176: 753–60.
[28] Frith P, Crockett A, Beilby J, et al. Simplified COPD screening: validation of the PiKo-6® in primary care. Prim Care Respir J. 2011. 20(2): 190-8, 2 p following 198.
[29] Labor M, Vrbica Ž, Gudelj I, Labor S, Plavec D. Diagnostic accuracy of a pocket screening spirometer in diagnosing chronic obstructive pulmonary disease in general practice: a cross sectional validation study using tertiary care as a reference. BMC Fam Pract. 2016. 17(1): 112.
[30] Stanley AJ, Hasan I, Crockett AJ, van Schayck OC, Zwar NA. COPD Diagnostic Questionnaire (CDQ) for selecting at-risk patients for spirometry: a cross-sectional study in Australian general practice. NPJ Prim Care Respir Med. 2014. 24: 14024.
[31] Zhang Q, Wang M, Li X, Wang H, Wang J. Do symptom-based questions help screen COPD among Chinese populations. Sci Rep. 2016. 6: 30419.
[32] A Guide to Performing Quality Assured Diagnostic Spirometry. http://www.artp.org.uk/en/professional/artp-tandards/index.cfm/QADS%20Apr%202013.
[33] Alonzo TA, Pepe MS, Moskowitz CS. Sample size calculations for comparative studies of medical tests for detecting presence of disease. Stat Med. 2002. 21(6): 835-52.
[34] Represas-Represas C, Fernández-Villar A, Ruano-Raviña A, Priegue-Carrera A, Botana-Rial M. Screening for Chronic Obstructive Pulmonary Disease: Validity and Reliability of a Portable Device in Non-Specialized Healthcare Settings. PLoS One. 2016. 11(1): e0145571.
[35] Van den Bemt L, Wouters BC, Grootens J,et al. Diagnostic accuracy of pre-bronchodilator FEV1/FEV6 from microspirometry to detect airflow obstruction in primary care: a

For peer review only

randomised cross-sectional study. NPJ Prim Care Respir Med. 2014 Aug 14;24:14033.
[36] Medium-long term plan of prevention and treatment for chronic diseases (2017-2025) http://www.gov.cn/xinwen/2017-02/14/content_5167942.htm.(in Chinese)

For peer review only

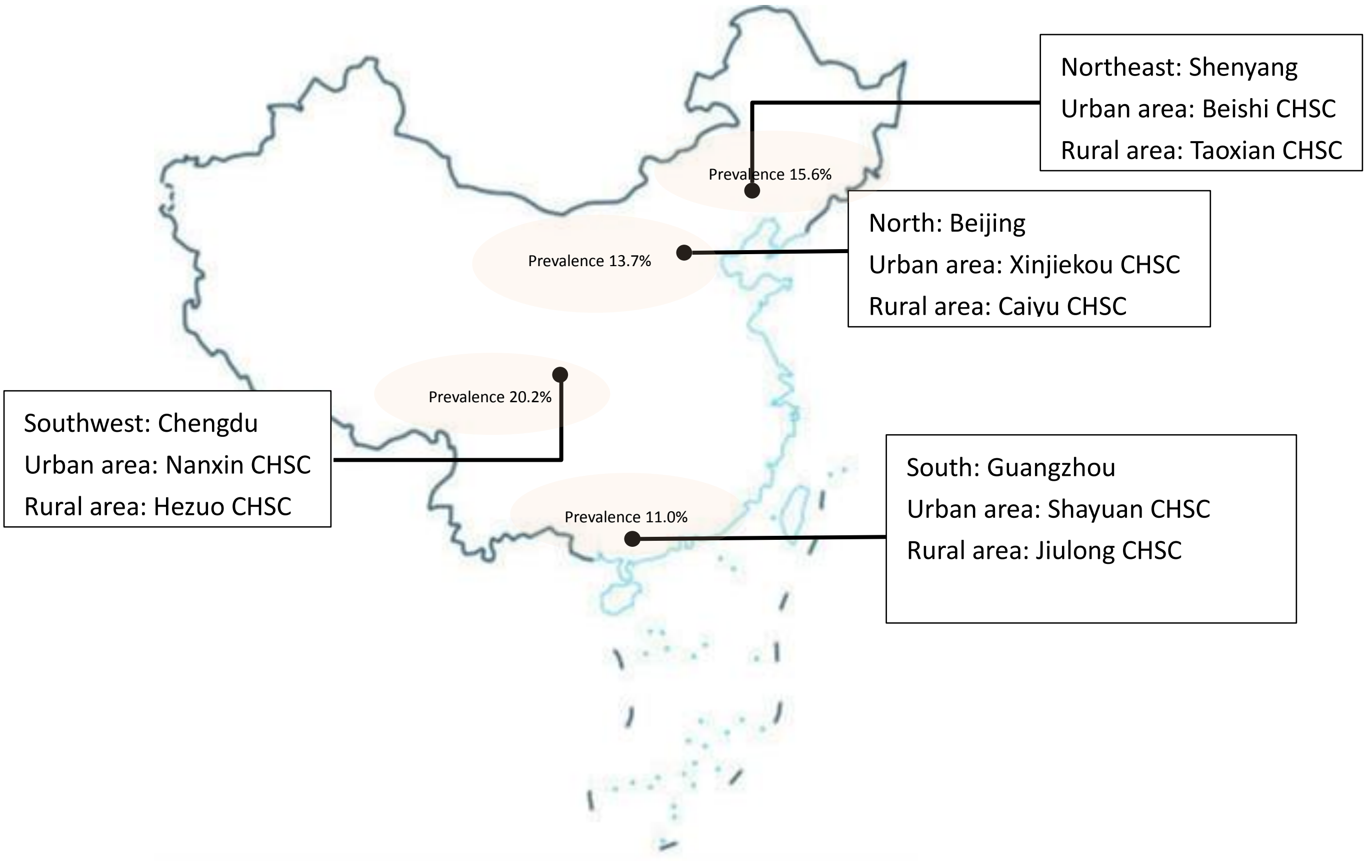

Figure 1 the map of Breathe Well-China research sites

For peer review only

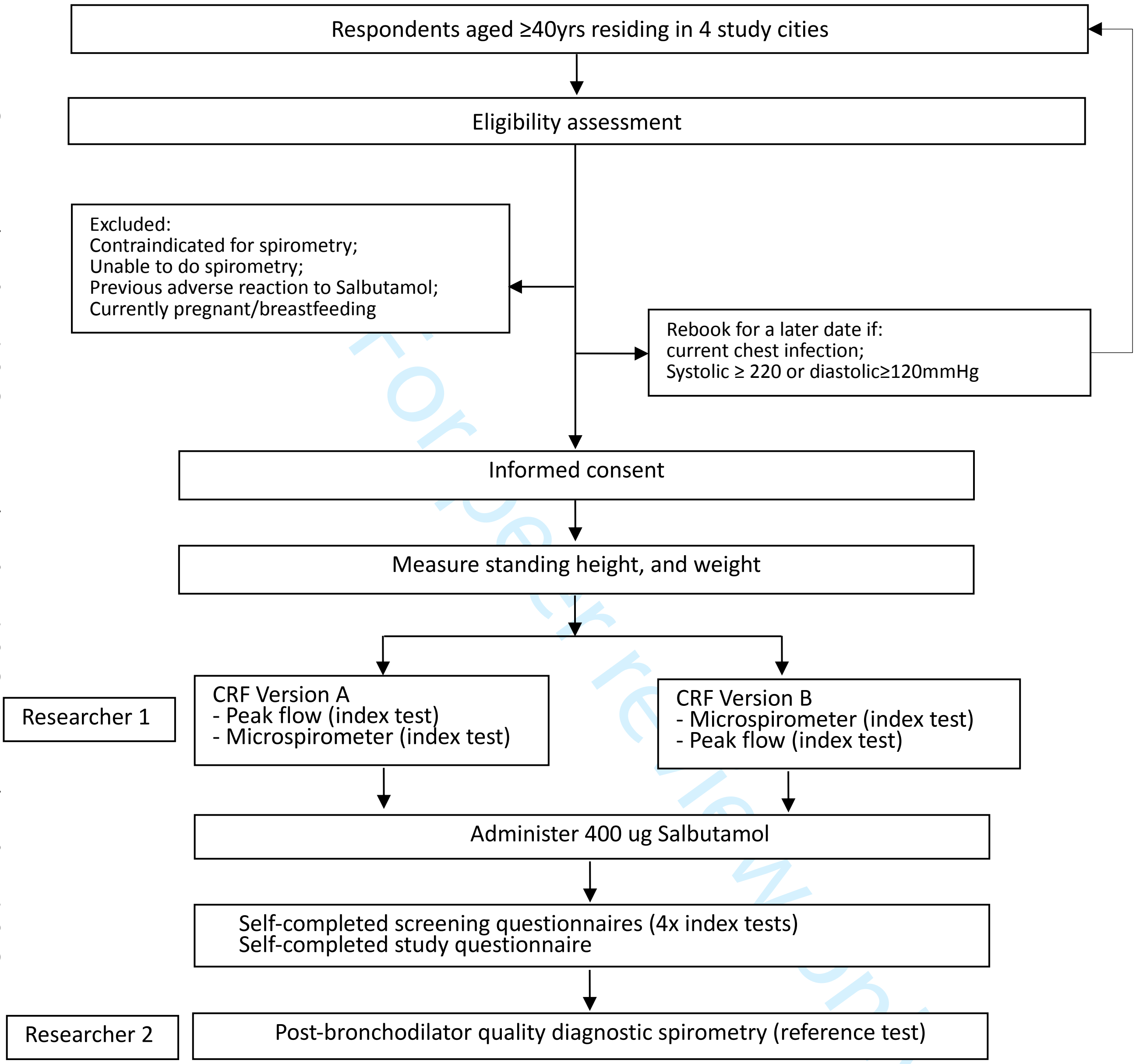

Figure 2 flow of participants

Evaluating screening strategies for identifying undiagnosed COPD in China: a Breathe Well project

中国慢阻肺筛查策略评估：健康呼吸 Breathe Well 研究项目

For peer review only

Lung health questionnaire

肺部健康问卷

Participant Initials<br>研究对象编号	
Study ID<br>问卷编号	
Date<br>填写日期	
Interviewer ID<br>研究人员编号	

For peer review only - http://bmjopen.bmj.com/site/about/guidelines.xhtml

筛查问卷 版本号：1.0 版本日期：2018.5.9

Some questions in the following booklets may appear similar. However, it is important that we ask these questions in slightly different ways so please complete all questions, answering them as accurately as possible.

一些问题可能相似，但是我们以稍微不同的方式提出这些问题很重要。

因此，请您完成所有的问题，并尽可能准确地作答。

CDQ

1. Age group, years
   年龄

40–49 ☐ 50-59 ☐ 60-69 ☐ 70+ ☐

2. What is your weight in kilograms?
   您的体重（公斤）？

   ______________kilograms
   ______________ 公斤

   What is your height in meters?
   您的身高（米）？

   ______________ metres
   ______________ 米

3. Smoking
   吸烟强度，包年
   What is the total number of years you have smoked?
   您一共吸烟多少年？
   _____ years
   _____ 年

   How many cigarettes do you currently smoke each day (or 'did smoke each day' if ex-smoker)?
   目前您每天吸多少支烟？（或，如果是既往吸烟者，过去您每天吸多少支烟？）
   _____ cigarettes
   _____ 支

4. Does the weather affect your cough?
   您的咳嗽是否受天气影响？

Yes ☐ No ☐

For peer review only

是 ☐ 否 ☐

5. Do you ever cough up phlegm (sputum) from your chest when you don't have a cold?
   您不感冒的时候，会从胸腔里咳出痰吗？（区别于从嗓子中咳痰）

Yes ☐ No ☐
是 ☐ 否 ☐

6. Do you usually cough up phlegm (sputum) from your chest first thing in the morning?
   清晨您的第一件事是从胸腔里咳出痰吗？

Yes ☐ No ☐
是 ☐ 否 ☐

7. How frequently do you wheeze?
   您喘息的次数是多少？

Occasionally or more often ☐ Never ☐
有时候或更频繁 ☐ 从不 ☐

8. Do you have or have you had any allergies?
   目前或既往您有过敏物吗？

Yes ☐ No ☐
是 ☐ 否 ☐

CAPTURE

1. Have you ever lived or worked in a place with dirty or polluted water or air, smoke or second-hand smoke or dust?
   您是否曾经在有脏的或受到污染的水或空气，烟雾或二手烟雾或灰尘的地方生活或工作？

Yes ☐ No ☐
是 ☐ 否 ☐

2. Does your breathing change with seasons, weather or air quality?
   您的呼吸是否随着季节、天气或空气质量而变化？

Yes ☐ No ☐
是 ☐ 否 ☐

3. Does your breathing make it difficult to do things such as carry heavy loads, shovel dirt or snow, jog, play tennis or swim?
   您的呼吸是否会使您难以进行一些工作，比如提重物，铲土或积雪，慢跑，打网球或游泳等？

For peer review only

Yes ☐ No ☐
是 ☐ 否 ☐

4. Compared to others your age, do you tire easily?
和您的同龄人相比，您是否容易感到疲劳？

Yes ☐ No ☐
是 ☐ 否 ☐

5. In the past 12 months, how many times did you miss work, school, or other activities due to a cold, bronchitis, or pneumonia?
在过去的 12 个月里，您有多少次因感冒、支气管炎或肺炎而错过了工作、学校或其他活动？

0 ☐ 1 ☐ 2 or more ☐
0 ☐ 1 ☐ 2 或以上 ☐

Copyright© 2015 by Cornell University, University of Kentucky, and Evidera. All Rights Reserved
版权所有©2015 康奈尔大学，肯塔基大学和 Evidera。版权所有

Symptom-based questionnaire

1. How frequently are you exposed to second-hand smoking?
您接触二手烟的频率是多少？

<7hrs per week ☐ ≥7hrs per week ☐
＜7小时/周 ☐ ＞7 小时/周 ☐

2. Do you often cough when you do not have a cold?
您是否在不感冒的时候经常咳嗽？

Yes ☐ No ☐
是 ☐ 否 ☐

3. Do you have more signs of shortness of breath compared with others of the same age?
和同龄人相比，您是否有更多的呼吸急促的症状？

Yes ☐ No ☐
是 ☐ 否 ☐

4. Have you had long-term exposure to dust or chemical particles?
您是否长期地接触粉尘或化学颗粒？

Yes ☐ No ☐

For peer review only - http://bmjopen.bmj.com/site/about/guidelines.xhtml

是 ☐ 否 ☐

5. Did you have a history of chronic respiratory diseases when you were a child?
   在您孩童时期，您是否有慢性呼吸疾病的病史？

Yes ☐是 No ☐
是 ☐ 否 ☐

COPD-SQ

1. Do you often cough?
   您是否经常咳嗽？

Yes ☐ No ☐
是 ☐ 否 ☐

2. Family history of respiratory disease
   是否有呼吸疾病家族史？

Yes ☐ No ☐
是 ☐ 否 ☐

3. Exposure to biomass smoke from cooking fires
   是否接触烹饪产生的生物烟雾？

Yes ☐ No ☐
是 ☐ 否 ☐

For peer review only

中国慢阻肺筛查策略评估：健康呼吸 Breathe Well 研究项目

Evaluating screening strategies for identifying undiagnosed COPD in China: a Breathe Well project

调查问卷

Study Questionnaire

研究对象编号<br>Patient Initials	
问卷编号<br>Study ID	
填写日期<br>Date	
研究人员编号<br>Interviewer ID	

For peer review only

您的回答和意见对们很有价值。请您在翻页之前阅读以下内容，非常感谢您的合作！

Your answers and opinions are valuable to us. We would be very grateful if you could read the below before turning the page:

- **如有可能，请您自行填写这份问卷。**

  Please complete this questionnaire yourself if at all possible
- **请尽可能回答所有问题**

  Please answer all questions as well as you can
- **请不要花太多时间思考您的回答**

  Do not spend too long thinking about your answers
- **如果有人替您回答了这份问卷，他们需要记录下您的答案**

  If someone is completing this on your behalf, they should record your answers

For peer review only - http://bmjopen.bmj.com/site/about/guidelines.xhtml

1. 性别

Sex

☐ 男

Male

☐ 女

Female

2. 年龄

What is your age?

岁

___________ years old

3. 您获得的最高学历是什么？

What is the highest level of qualification that you have?

没有正式的学历 ☐

No formal qualification

低于高中水平 ☐

Less than High school

高中水平 ☐

High school

大专

Junior college

本科 ☐

Bachelor

研究生 ☐

Master

博士 ☐

Doctor

4. 您的工作状态是什么样的？

What is your employment status?

调查问卷 v7 2018.10.4

Study questionnaire 041018

For peer review only

个体
Self-employed ☐

受雇于工作单位
Employed ☐

无工作
Unemployed ☐

退休
Retired ☐

5. 您绝大部分时间生活在哪里？
Where have you spent most of your life?

城市
Urban areas ☐

农村
Rural areas ☐

6. 您目前的吸烟状态是？
What is your current smoking status?

当前吸烟者（每天至少吸 1 支，至少吸了 6 个月）
Current smoker (smoke at least 1 cigarette per day for at least the last 6 months) ☐

既往吸烟者（既往每天至少吸 1 支，至少吸了 6 个月，但是现在不吸了）
Ex-smoker (previously smoked at least 1 cigarette per day for at least 6 months, but not now) ☐

我从不经常性地吸烟 （**如果您选择了这个选项，请跳至第 9 题**）
I have never smoked regularly （***please go to question 10***） ☐

7. 如果您曾经吸过烟，那么您是几岁**开始**经常性地吸烟？（“经常性地吸烟”指的是，至少 1 支/每天或者 7 支/每周，至少 6 个月）
If you have ever smoked, at about what age did you **start** to smoke regularly? (by regularly we mean at least 1 cigarette/day or 7 cigarettes/week for at least 6 months)
________ 岁

For peer review only - http://bmjopen.bmj.com/site/about/guidelines.xhtml

For peer review only

_________ years old

如果您曾经吸过烟，您是从什么时候**停止**经常性地吸烟的？
If you are an ex-smoker, at what age did you **stop** smoking regularly?
_________ 岁
_________ years old

8．目前您每天常常吸多少支烟？或者，当您是烟民的时候，您是否经常性地吸烟？
How much do you usually smoke each day now, or did you usually smoke when you were a smoker?

电子烟<br>Electronic cigarettes (or e-cigarettes)		支/天<br>number/day
过滤嘴型香烟<br>Filter cigarettes		支/天<br>number/day
无过滤嘴/手卷烟<br>Non-filter/hand rolled cigarettes		支/天<br>number/day
雪茄<br>Cigars		支/天<br>number/day
烟斗<br>Pipe tobacco		烟草…克/天<br>g/day tobacco

9．您的整体健康状况如何?
How is your health in general?
非常好 Very Good☐ 好 Good☐ 一般 Fair☐ 差 Bad☐ 非常差 Very Bad☐

10．患病情况
Medical conditions

您患有以下疾病吗？请选择
Has a doctor EVER told you that you had any of the following conditions? Please tick all that apply

疾病<br>Conditions	有<br>Yes	无<br>No
慢性阻塞性肺疾病<br>Chronic Obstructive Pulmonary Disease		
慢性支气管炎/肺气肿<br>Chronic bronchitis/emphysema		

调查问卷 v7 2018.10.4
Study questionnaire 041018

For peer review only

哮喘<br>Asthma		
结核<br>Tuberculosis		
高血压<br>Hypertension		
糖尿病<br>Diabetes Mellitus		
胃食管返流<br>GERD		
焦虑<br>Anxiety		
抑郁<br>Depression		
心脏病<br>Heart disease		
癌症<br>Cancer		

11. 当您在水平地面上行走或在一个小山坡上行走时，您是否因呼吸急促而感到困扰？

Are you troubled by shortness of breath when hurrying on the level ground or walking up a slight hill?

是 Yes ☐ 否 No ☐

12. 您在平地上和同龄人一起行走时，您是否会感到气促？

Do you get short of breath walking with other people of your own age on level ground?

是 Yes ☐ 否 No ☐

13. 当您在平地上按自己的速度行走时，您是否会因为呼吸而不得不停下来？

Do you have to stop for breath when walking at your own pace on level ground?

是 Yes ☐ 否 No ☐

14. 当您在平地上行走 100 米或几分钟后，您是否会因为呼吸而不得不停下来？

Do you have to stop for breath after walking for 100yds (or after a few minutes) on the level?

是 Yes ☐ 否 No ☐

15. 您是否因呼吸困难而不能离开家或者您是否在穿衣服或脱衣服的时候有呼吸困难？

Are you too breathless to leave the house or are you breathless when dressing or undressing?

是 Yes ☐ 否 No ☐

For peer review only - http://bmjopen.bmj.com/site/about/guidelines.xhtml

COPD 评估 (CAT)

COPD Assessment Test (CAT)

即使您没有肺部问题，也请完成以下问卷。

Please complete the below questionnaire even if you do not have a lung condition

16. 您肺部的问题怎么样？对于下面的每个项目，请在 0-5 中圈出最符合您的情况的分数。

How are your lung problems? For each item below place a mark in the box that best describes your experience on a scale of 0-5

例如：我极开心	0	✓ 1	2	3	4	5	我极不开心

**Example:** I am very happy	0	√ 1	2	3	4	5	I am very sad

我从不咳嗽<br>I never cough	0	1	2	3	4	5	我总是咳嗽<br>I cough all the time
我肺里一点痰也没有<br>I have no phlegm (mucus) in my chest at all	0	1	2	3	4	5	我肺里有很多很多痰<br>My chest is completely full of phlegm (mucus)
我一点也没有胸闷的感觉<br>My chest does not feel tight at all	0	1	2	3	4	5	我有很重的胸闷的感觉<br>My chest feels very tight
当我在爬坡或爬一层楼时，我并不感觉喘不过气来<br>When I walk up a hill or one flight of stairs I am not breathless	0	1	2	3	4	5	当我爬坡或爬一层楼时，我感觉非常喘不过气来<br>When I walk up a hill or one flight of stairs I am very breathless
我在家里的任何劳动都不受慢阻肺的影响<br>I am not limited doing any activities at home	0	1	2	3	4	5	我在家里的任何劳动都很受慢阻肺的影响<br>I am very limited doing activities at home
尽管我有肺病，我还是有信心外出<br>I am confident leaving my home despite my lung condition	0	1	2	3	4	5	因为我有肺病，对于外出我完全没有信心<br>I am not at all confident leaving my home because of my lung condition

调查问卷 v7 2018.10.4
Study questionnaire 041018

For peer review only - http://bmjopen.bmj.com/site/about/guidelines.xhtml

我睡得好<br>I sleep soundly	0 1 2 3 4 5	因为我有肺病，我睡得不好<br>I don't sleep soundly because of my lung condition
我精力旺盛<br>I have lots of energy	0 1 2 3 4 5	我一点精力都没有<br>I have no energy at all

COPD 评估测试和 CAT 的标志是 GlaxoSmithKline 集团公司的商标。
©2009 GlaxoSmithKline 集团公司。版权所有。

17．您在儿童时期，患过气管炎，肺炎或者严重的百日咳吗？
Did you ever have bronchitis, pneumonia or severe whooping cough as a child?

☐ 有
Yes

☐ 无 如果没有，跳至第 19 题
No If no, ***please go to question 19***

18．如果有，您患病的时候多大？（或者首次发作的时候）？
If yes, approximately how old were you when you had this (or first time if several episodes)?

___岁
_____years
或
or
______月
_____months

19．您在孩童时期，是否患过肺结核？
Did you ever have tuberculosis as a child?

☐ 是
Yes

☐ 否，如果否，请跳至第 21 题
No If no, ***please go to question 21***

20．如果是，那时您大概多大？（或者第一次患病多大，如果有复发的话）
If yes, approximately how old were you when you had this (or first time if several episodes)?

____年
_____years
或
Or
______月
______months

For peer review only - http://bmjopen.bmj.com/site/about/guidelines.xhtml

21. 以下哪些化学物质或颗粒是您目前正在工作/家中接触的，或者您在工作/家中已经接触过了哪些？(生物质燃料包括木柴，粪肥，农作物残留物如秸秆/草/灌木，煤和煤油)

Which of the following chemicals or particulates are you currently exposed to at work/home, or which have you been exposed to at work/home in the past? (Biomass fuel consists of fire wood, manure, agricultural crop residues such as straw/grass/shrubs, coal fuels and kerosene)

物质种类<br>chemicals or particulates	是，正在接触<br>Yes, currently	是，过去接触过<br>Yes, in the past	否，从没接触过<br>No, never
烹饪油烟<br>Cooking fumes			
生物质燃料<br>Biomass fuel			
各种物质的蒸汽<br>Steam of various substances			
气体<br>Gas			
灰尘<br>Dust			

22. 如果您接触过上述物质，您接触了多少年？

If you ticked 'yes' to any exposures, how many years have you been exposed to them?

________年

_______years

23. 如果您接触了烹饪油烟或者生物质燃料，您的家中/工作地点有烟囱或排烟系统吗？

If exposed to cooking fumes or biomass fuels, did the home/workplace have a chimney or exhaust system?

☐ 有

Yes

☐ 无

No

非常感谢您抽出宝贵的时间参与本研究！

Thank you for taking the time to complete this survey

调查问卷 v7 2018.10.4

Study questionnaire 041018

For peer review only

Study ID ☐☐☐☐☐☐

COPD case finding study: assessment of task timing

IMPORTANT: Please write how long each task takes in minutes.

Assessment station 1 – NO TIMING REQUIRED

Assessment station 2

Please only note the time for standing height (not arm span or weight)

Standing height **start time ______ end time ______** ☐☐ minutes

Assessment station 3

Pre-bronchodilator peak flow **start time ______ end time ______** ☐☐ minutes

Pre-bronchodilator microspirometry **start time ______ end time ______** ☐☐ minutes

Assessment station 4

Administration of Salbutamol **start time ______ end time ______** ☐☐ minutes

Assessment station 5

Completion of Lung Health questionnaire (CDQ etc) **start time ______ end time ______**

☐☐ minutes

Did the patient require assistance? Yes ☐ No ☐

If yes, was assistance required for the whole questionnaire? Yes ☐ No ☐

Assessment station 6

Post-bronchodilator spirometry **start time ______ end time ______** ☐☐ minutes

For peer review only

北京大学第一医院生物医学研究伦理委员会审查批件

伦理审查编号：(2018)科研第(141)号-修正案　　EC 存档档案号：2018 研 141

伦理委员会批准日期：2018 年 11 月 07 日　批件有效期至：2019 年 11 月 06 日　定期跟踪审查频率：12 个月

项目名称	慢性阻塞性肺疾病不同筛查策略在中国四个城市≥40 岁人群中的有效性及成本-效益的评价性研究-横断面研究		
CFDA 批件号	/		
申办者	北京大学第一医院　健康管理中心		
临床研究科室	健康管理中心	主要研究者	迟春花
批准的文件	1、研究方案（版本号：6　版本日期：2018.10.25）<br>2、知情同意书（版本号：6.0　版本日期：2018.10.25）<br>3、研究对象信息表（版本号:v1　版本日期：2018.10.19）<br>4、肺部健康问卷（版本号：1.0　版本日期：2018.5.9）<br>5、调查问卷（版本号：v7　版本日期：2018.10.4）<br>6、中国案例报告表（版本号：Version3　版本日期：2018.10.25）		
**本伦理委员会的人员组成和工作程序符合中国 GCP 以及国家相关规定**			
伦理审查方式：■快速审查　审查时间：2018 年 11 月 07 日			
审查委员	张宝娓　谢鹏雁		
审查意见	同意按照上述批准的文件进行该临床试验。		

注意事项：

1. 本项临床试验应当在伦理委员会同意进行之日起 1 年内实施。逾期未实施的，本审查批件自行废止。
2. 研究应遵循本伦理委员会批准的方案执行，须符合 GCP 和《赫尔辛基宣言》的原则。
3. 自同意研究之日起，每隔 12 个月伦理委员会的定期跟踪审查（审查频度可能根据实际进展情况改变）；请在定期跟踪审查到期前 1 个月递交《定期跟踪审查表》。
4. 研究过程中，对研究方案和知情同意书等相关文件所作的任何修改，请交《修正案申请表》及“送审文件清单”中规定相关资料，并得到伦理委员会审查同意该修正后方可实施。
5. 发生严重不良事件或影响研究风险受益比的非预期不良事件，在向 CFDA 上报的同时向伦理委员会作书面通报，可以使用 CFDA 的《严重不良事件报告表》或本伦理委员会公布的《严重不良事件/非预期不良事件报告表》或其他有相关内容的报告表，但外文的报告需要有中文摘要。伦理委员会有权根据对其评估做出新的决定。
6. 不依从或违反方案应及时提交《不依从或违反方案报告表》。
7. 提前终止研究应及时提交《研究方案提前终止报告表》。
8. 研究完成后提交《研究结题报告表》和临床试验总结报告。
9. 及时书面报告其他伦理委员会的重要决定。

主任委员或副主任委员签名：[illegible]

北京大学第一医院生物医学研究伦理委员会（盖章）

2018 年 11 月 7 日

伦理委员会地址：北京市西城区大红罗厂街 6 号　邮编：100034　联系电话：010-66119025

发件人: Susan Cottam <s.l.cottam@bham.ac.uk>
发送时间: 2018年10月22日星期一 17:30
收件人: Andy Dickens
主题: Application for Ethical Review ERN_18-1177

Dear Dr Dickens

Re: "A study to evaluate the effectiveness and cost-effectiveness of different screening strategies for identifying undiagnosed COPD in China, amongst residents (≥ 40 years) in four cities"
Application for Ethical Review ERN_18-1177

Thank you for your application for ethical review for the above project, which was reviewed by the Science, Technology, Engineering and Mathematics Ethical Review Committee.

On behalf of the Committee, I confirm that this study now has full ethical approval.

I would like to remind you that any substantive changes to the nature of the study as described in the Application for Ethical Review, and/or any adverse events occurring during the study should be promptly bought to the Committee's attention by the Principal Investigator and may necessitate further ethical review.

Please also ensure that the relevant requirements within the University's Code of Practice for Research and the information and guidance provided on the University's ethics webpages (available at https://intranet.birmingham.ac.uk/finance/accounting/Research-Support-Group/Research-Ethics/Links-and-Resources.aspx) are adhered to and referred to in any future applications for ethical review. It is now a requirement on the revised application form (https://intranet.birmingham.ac.uk/finance/accounting/Research-Support-Group/Research-Ethics/Ethical-Review-Forms.aspx) to confirm that this guidance has been consulted and is understood, and that it has been taken into account when completing your application for ethical review.

Please be aware that whilst Health and Safety (H&S) issues may be considered during the ethical review process, you are still required to follow the University's guidance on H&S and to ensure that H&S risk assessments have been carried out as appropriate. For further information about this, please contact your School H&S representative or the University's H&S Unit at healthandsafety@contacts.bham.ac.uk.

Kind regards

Susan Cottam
Research Ethics Officer
Research Support Group
C Block Dome
Aston Webb Building
University of Birmingham
Edgbaston B15 2TT
Tel: 0121 414 8825
Email: s.l.cottam@bham.ac.uk
Web: https://intranet.birmingham.ac.uk/finance/RSS/Research-Support-Group/Research-Ethics/index.aspx

Please remember to submit a new Self-Assessment Form for each new project.

You can also email our team mailbox ethics-queries@contacts.bham.ac.uk with any queries relating to the University's ethics process.

For peer review only - http://bmjopen.bmj.com/site/about/guidelines.xhtml

Click Research Governance for further details regarding the University's Research Governance and Clinical Trials Insurance processes, or email researchgovernance@contacts.bham.ac.uk with any queries relating to research governance.

Notice of Confidentiality:
The contents of this email may be privileged and are confidential. It may not be disclosed to or used by anyone other than the addressee, nor copied in any way. If received in error please notify the sender and then delete it from your system. Should you communicate with me by email, you consent to the University of Birmingham monitoring and reading any such correspondence.

For peer review only

For peer review only - http://bmjopen.bmj.com/site/about/guidelines.xhtml

Evaluating screening strategies for identifying undiagnosed COPD in China: a Breathe Well project

Informed consent

We are conducting a study to evaluate the accuracy of different screening methods for chronic obstructive pulmonary disease (COPD) in people aged 40 years and older. We are inviting you to participate in this study because your condition meets the criteria for the enrolment. This sheet will give you a brief introduction for the purpose, process, benefits, and risks of this study. Please read it carefully before deciding whether you are interested in taking part, and you are welcome to discuss it with your family and friends. When the researcher explains and discusses the informed consent form, you can ask questions and ask him/her to explain anything to you if you have anything you don't understand. You can make a decision after discussing with your family, friends, and your doctor.

The study is funded through a collaboration with the University of Birmingham in the UK The primary investigator of this study is Chi Chunhua, chief physician of Peking University First Hospital.

1. Why do we conduct this study?

COPD has brought a huge economic burden to the country and has also brought about psychological and economic hardships for patients. China has a large population of patients with COPD. However, it faces the problems of high rate of missed and late diagnosis. The community is an important checkpoint for the prevention and treatment of chronic non-communicable diseases, and the community has unique advantages in early detection and early intervention in the COPD population. However, at present, what is cost-effective screening method for early detection of COPD patients is not clear. In view of this, we designed this study to assess the best screening methods for COPD in the community in China. The test commonly used to assess people's lung health is called spirometry, and this will be compared against screening tests including a peak flow meter, microspirometry and questionnaires.

2. Who will be invited to participate in this study?

For peer review only

Eligibility criteria:

Inclusion

- Aged ≥40 years
- Residing in the catchment areas

Exclusion

- Unable to do spirometry (e.g. dementia, lack of teeth-cannot make a good seal)
- Contraindicated for spirometry (chest infection in the last 3 weeks, coughing up blood in the last month, severe angina, uncontrolled high blood pressure, pneumothorax or history in the last 3 months of tuberculosis, heart attack, detached retina, or surgery on chest/abdomen/brain/ears/eyes
- Currently pregnant/breastfeeding
- Previous adverse reaction to Salbutamol

In addition, the researcher will judge according to your actual situation whether you are suitable for this study.

3. How many people will be recruited in this study?

We are recruiting approximately 2,000 subjects in the study, each community health centres need to recruit nearly 250 subjects.

4. What is the process of the study?

This study is a multi-centre cross-sectional survey (diagnostic test accuracy study). The research sites are Beijing (North), Chengdu (Southwest), Guangzhou (South), and Shenyang (Northeast). Each city selects two research sites: a community health service centre in urban area, and another in rural area.

Instruments model and drug information:

Instruments:

1) Expiratory peak flow meter(PEF): USPE
2) Microspirometer: COPD-6
3) Spirometer: ndd Easy On-PC (ndd Medizintechnik AG)

Funded by NIHR

For peer review only - http://bmjopen.bmj.com/site/about/guidelines.xhtml

Drug:

Bronchodilator: Salbutamol Aerosol (GSK)

The instruments and drugs used in this study have been approved by the State Food and Drug Administration (CFDA).

Study assessment visit:

If you participate in the study, you would attend one study assessment visit lasting approximately 90 minutes in total. If you are eligible for the study you will have your height and weight measured, before being asked to do three blowing tests and complete study questionnaires.

The data collection process is as flows:

Assessment station 1:

The researcher will confirm your eligibility for the study based on the criteria mentioned above and check for current chest infection (those with acute infections will be rebooked). If you are eligible you will then have opportunity to ask any questions about the study before completing the consent form with the researcher. As well as consenting to the main study, you will be asked to consent to being contacted about future research related to the study. Future contact will be optional and will not affect your ability to take part in the study.

Assessment station 2:

The researcher will measure your height and weight.

Assessment station 3:

You will be asked to perform blowing tests on two different devices, one called a peak flow meter and one called a microspirometer. You will be asked to perform 3 blows on each device, and further details are given below.

Lung function (peak flow)

Funded by NIHR

A trained researcher will assess peak expiratory flow using a simple peak flow meter (USPE). For the main analysis, peak expiratory flow rates (PEFR) of <350 l/min for men and <250 l/min for women will be used to indicate a positive test Standardized researcher training will detail how to perform the technique and how to use the device. Each participant will perform three blows without administration of salbutamol, after which the researcher will record the highest PEF.

Lung function (microspirometry)

Microspirometry will be performed with minimal coaching by a trained researcher using a simple handheld microspirometer (COPD6), to measure FEV_1, FEV_6 and FEV_1/FEV_6 ratio. Microspirometer devices will be checked for calibration errors at the start of the study by the researchers. Standardized researcher training will detail how to perform the technique and how to use the device. Each participant will perform three blows using the device, after which the researcher will record the highest FEV_1 and FEV_6 values and the FEV_1/FEV_6 ratio. For the main analysis, FEV1/FEV6 ratios of <0.75 and <0.78 will be used to indicate a positive test.

Assessment station 4:

You will receive 400 µg of salbutamol using a large volume spacer, and then wait approximately 20 minutes before performing another blowing test.

Assessment station 5:

During the 20 minutes waiting period, you will be asked to complete study questionnaires by yourself, in a separate area of the assessment clinic, and return the completed forms to the researcher. The study pack will include four screening questionnaires (CAPTURE, CDQ, COPD-SQ and a symptom-based questionnaire). In addition to the screening questionnaires, the study pack will also include items relating to the following topics: demographic data (sex, age, marital status, education level, deprivation); lifestyle (smoking status, exercise); exposures (biomass smoke,

For peer review only

occupational exposure to chemicals and particulates); health (medical diagnoses, comorbidities, respiratory symptoms, use of medications); quality of life (COPD assessment test (CAT)).

A member of the research team will be available to help you complete questionnaires if necessary.

Assessment station 6:

After completing the questionnaires, you will be asked to perform another blowing test using a portable spirometer (ndd Easy On-PC) that will be linked to a laptop. You may be asked to repeat this blowing test up to a maximum of 6 times.

5. How long will this study last?

This study will last for approximately one year.

6. What are the risks of participating in this study?

Blowing tests will be performed multiple times depending on the research needs. Multiple examinations may cause discomfort to some patients. If any discomfort occurs, please inform the researchers at any time. We will give you Salbutamol before the final blowing test. There is a very small risk of drug allergy. If you have adverse reactions with salbutamol in this study, please be sure to inform the investigator. Possible side effects from Salbutamol are rare and include feeling shaky, rapid heart rate or headache. If you experience any of these, it will be immediately after taking Salbutamol and will disappear after several minutes

Some questions in the questionnaire may make you feel uncomfortable, if so, ,you can refuse to answer.

7. What are the benefits of participating in this study?

You will not benefit directly from participating in this study. Your participation will help us find an effective, cost-effective method of screening for COPD, so that patients with COPD can be detected early, and they can be treated promptly to relieve their condition.

For peer review only

At the end of the study assessment, researchers will explain your lung function results to you and answer any immediate questions you may have.

8. Is it necessary to participate in and complete this study?

It is entirely voluntary whether you participate in this research. If you do not want to, you can refuse to participate, which will not have any negative impact on your current or future medical care. Even if you agree to participate, you can change your mind at any time and tell the researchers to withdraw from the study. Your withdrawal will not affect your access to normal medical services.

If you decide to withdraw from this study, we will stop collecting new data related to this study from you.

9. Fees and compensation for participating in the study

You don't need to pay any cost to participate in this study.

We will compensate you for your time and support for participation by giving you small household tokens (e.g. washing powder, toothpaste, liquid soap).

This study does not provide monetary compensation such as transportation fees and losing of working time.

10. What happened to research-related injuries?

There are no relevant invasive tests in this study.

If you have any discomfort during the research process, please inform the researchers. We will promptly take the necessary medical measures for treatment or inform the researcher (main researcher **Pan Zihan**, Tel. **18701291196**). If it is confirmed that the health status is harmed because of participating in this study, you'll be compensated according to the current laws.

11. Is my information confidential?

If you decide to participate in this study, your participation in the study and personal data are confidential. Information that identifies you will not be disclosed to anyone other than the research member unless we have your permission. All research members are required to keep your identity confidential. Your file will be kept in a

For peer review only

locked file cabinet for research purposes only. In order to ensure that the research is conducted in accordance with the regulations, members of the government management department or ethics committee may, as required, consult your personal data in the research sites.

12. Data storage

Data will be stored securely at the community centre, the co-ordinating study centre and on an online database that is held on servers at the University of Birmingham, UK. For further information, please refer to the Breathe Well website (https://www.birmingham.ac.uk/breathewell).

13. Publication of study findings

When we have analysed the results we will publish them in an academic journal. All publications will be available on the Breathe Well website. You will not be identified in any publication.

14. Who should I contact if I have problems or difficulties?

If you have any problems or difficulties related to this study, please contact **Dr. Pan Zihan**, her telephone number is **18701291196**.

If you have any problems or difficulties related to the subject's own rights, you can contact the Biomedical Research Ethics Committee of the **Peking University First Hospital** ,Tel: **010-66119025**.

15. Funding

The research was commissioned by the National Institute for Health Research using Official Development Assistance (ODA) funding. The views expressed are those of the author(s) and not necessarily those of the NHS, the NIHR or the Department of Health.

16. Signature

Study ID: ________________

Subject statement

The investigators explained to me the background, purpose, process, risks, and benefits of participating in ”a study to evaluate the accuracy of different screening methods for chronic obstructive pulmonary disease（COPD）in people aged 40 years and older”. I have enough time and opportunity to ask questions. I am very satisfied with the answers provided by the researchers. I know who I should contact when I have questions or want to get further information. I read this informed consent and decided to participate in this study. I know that I can withdraw from this study at any time during the study without any reason. I was told I would get a copy of this informed consent, which contains the signature of me and the researcher.

I give my permission to be contacted in the future for related research purposes including other studies in the Breathe Well programme. ☐

Subject's signature: Date:

Legal representative's signature [if applicable]: Date:

Relationship with the subject:

Researcher's statement

I have explained to the subjects (and legal representatives) the background, purpose, process, risks, and benefits of participating in “a study to evaluate the accuracy of different screening methods for chronic obstructive pulmonary disease（COPD）in people aged 40 years and older”. He/she had enough time to read the informed consent form, discuss it with others, and we answered his/her questions about the study; I told the subject how to contact the persons when they have research-related questions; I told him/her (or legal representative) that he/she may withdraw this study without any reason at any time during the study.

Researcher's signature: Date:

Funded by NIHR
